# USLR: An open-source tool for unbiased and smooth longitudinal registration of brain MRI^☆^

**DOI:** 10.1016/j.media.2025.103662

**Published:** 2025-06-23

**Authors:** Adrià Casamitjana, Roser Sala-Llonch, Karim Lekadir, Juan Eugenio Iglesias

**Affiliations:** a Institut de Neurociències Department of Biomedicine, Faculty of Medicine, University of Barcelona, Barcelona, 08036, Spain; b Universitat Politècnica de Catalunya, Barcelona, 08034, Spain; c Institut d’Investigacions Biomèdiques August Pi i Sunyer (IDIBAPS), Barcelona, 08036, Spain; d Centro de Investigación Biomédica en Red de Bioingeniería, Biomateriales y Nanomedicina (CIBER-BBN), Barcelona, 08036, Spain; e Departament de Matemàtiques i Informàtica, Universitat de Barcelona, Artificial Intelligence in Medicine Lab (BCN-AIM), Barcelona, Spain; f Martinos Center for Biomedical Imaging, Massachusetts General Hospital and Harvard Medical School, United States of America; g Computer Science and Artificial Intelligence Laboratory, Massachusetts Institute of Technology, United States of America; h Centre for Medical Image Computing, University College London, United Kingdom; i Research Institute of Computer Vision and Robotics, University of Girona, Girona, 17003, Spain

**Keywords:** Unbiased longitudinal analysis, Smooth longitudinal registration, Subject-specific non-linear template, Tensor based morphometry, MRI biomarkers

## Abstract

We present the “Unbiased and Smooth Longitudinal Registration” (USLR) method, a computational framework for longitudinal registration of brain MRI scans to estimate non-linear image trajectories that are smooth across time, unbiased to any timepoint, and robust to imaging artefacts. It operates on the Lie algebra parameterisation of spatial transforms (which is compatible with rigid transforms and stationary velocity fields for non-linear deformation) and takes advantage of log-domain properties to solve the problem using Bayesian inference. USRL estimates spatial transformations that: (*i*) bring all timepoints to an unbiased subject-specific space; and (*ii*) compute a smooth trajectory across the imaging time-series. We capitalise on learning-based registration algorithms and closed-form expressions for fast inference. An Alzheimer’s disease study is used to showcase the benefits of the pipeline in multiple fronts, such as time-consistent image segmentation to reduce intra-subject variability, subject-specific prediction or population analysis using tensor-based morphometry. We demonstrate that such an approach improves upon cross-sectional methods in identifying group differences, which can be helpful in detecting more subtle atrophy levels or in reducing sample sizes in clinical trials. The code is publicly available in https://github.com/acasamitjana/uslr.

## 1. Introduction

Many of the central themes in neuroimaging, such as the effect of ageing, disease progression, or the effectiveness of a treatment, are intrinsically longitudinal. While cross-sectional studies are restricted to measuring population trajectories that may hide the true evolution of a given biomarker (Nyberg et al., 2010), longitudinal analysis uncovers truly individual trajectories that reduce confounding effects and dataset bias and results in better estimates — even in situations where population averages are aligned with the true effect (Maxwell and Cole, 2007; Kraemer et al., 2000). This increased power of longitudinal studies presents several opportunities, including: (*i*) better sensitivity and specificity, that could be used to detect different, partially overlapping atrophy patterns (Matuschek et al., 2017); (*ii*) reduced sample sizes for a target effect size (Naiji et al., 2013); and (*iii*) new surrogate endpoints for therapeutic interventions (Huang et al., 2012). Most importantly, longitudinal analysis produces individualised measures that are useful for a wealth of applications, such as post-treatment followup or monitoring of disease progression.

There exists a myriad of statistical models for data analysis that can appropriately handle longitudinal data, such as repeated measures ANOVA, linear mixed effects regression, or growth models, among others (Garcia and Marder, 2017). In the context of medical imaging, it is crucial to carefully design an image processing pipeline (e.g., spatial normalisation or segmentation) prior to feeding such statistical models with the appropriate data. However, most state-of-the-art brain image processing techniques are developed for cross-sectional settings and suffer from poor measurement reliability — which is a common limiting factor in longitudinal studies (Morey et al., 2010; Karch et al., 2019). Instead, longitudinal processing techniques are able to capture time dynamics and capitalise on the redundancy in the available repeated measures to produce a more consistent and reliable result. For example, subject-specific templates represent the “average” anatomy of a subject through time and can be used for longitudinal brain segmentation (Iglesias et al., 2016; Cerri et al., 2023). Similarly, groupwise registration techniques can be used for more reliable spatial normalisation to a subject-specific template (Joshi et al., 2004; Reuter et al., 2012). In conclusion, using an appropriate longitudinal processing followed by longitudinal statistical methods (e.g., linear mixed-effects model (LME) or repeated measures analysis of variance (ANOVA)) might produce more reliable results.

Nonetheless, longitudinal processing streams need to take into account several considerations. Primarily, uncorrelated sources of variability between timepoints, such as intensity inhomogeneities, subject motion and the appearance and evolution of brain lesions. All these are identified as major causes of error in atrophy estimation methods (Sharma et al., 2013). Likewise, longitudinal processing should be robust against intensity changes due to updates on the sequence, machine or scanning site during the follow up period as well as the inclusion of different MRI sequences, scanners, resolutions and field strengths between participants in multi-site studies (Lee et al., 2019). Recent works on domain randomisation are designed to tackle these issues in several applications, such as for image super-resolution (Iglesias et al., 2023), segmentation (Billot et al., 2023a) and registration (Hoffmann et al., 2022). Additionally, different sources of bias, such as interpolation asymmetries (i.e., using a given population template or choosing a timepoint as reference image) or excessive temporal regularisation, could hinder the power of the method or lead to false findings (Reuter et al., 2012; Thompson et al., 2011; Yushkevich et al., 2010).

The vast majority of classical existing registration algorithms, based on iterative numerical optimisation, are designed for pairwise alignment. Some examples are those implemented in widespread registration packages like NiftyReg (Modat et al., 2010), ANTs (Avants et al., 2008), Elastix (Klein et al., 2009), DARTEL (Ashburner, 2007), FNIRT (Andersson et al., 2007) or IRTK (Schnabel et al., 2001). Moreover, amidst the deep learning revolution, multiple learning based registration methods emerged both in supervised (Yang et al., 2017; Young et al., 2022) and unsupervised settings (De Vos et al., 2019). Again, most of these methods are trained using cross-sectional data. For example, the widespread Voxelmorph framework predicts a dense deformation field that registers pairs of T1w images independently (Balakrishnan et al., 2019). Its extension presented in Hoffmann et al. (2022) is able to register pairs of images of any contrast thus handling differences in scanners, sequences and protocols. Several frameworks for joint linear and non-linear learning-based registration have been introduced, such as DLIR (De Vos et al., 2019) or, more recently, EasyReg (Iglesias, 2023)

Many longitudinal studies are limited to 2 timepoints so that cross-sectional registration pipelines could be used. For example, in lesion follow-up studies, most often the baseline image is considered as reference as it usually contains smaller lesions (Diez et al., 2014; Dufresne et al., 2020). The work in Diez et al. (2014) compare different linear and non-linear registration methods to quantify the evolution of multiple sclerosis (MS) lesions. Seemingly, the authors in Dufresne et al. (2020) propose a joint model for registration and change detection in MS. In the context of radiotherapy, post treatment images are typically registered to the pre-treatment image for therapy follow-up (Lee et al., 2021, 2023). Lately, in the field of brain tumour resection (Baheti et al., 2021), several learning based approaches have been introduced, encouraging inverse consistency in the loss function during training (Wodzinski et al., 2022; Mok and Chung, 2022b) and handling missing correspondences between images (Mok and Chung, 2022a). Here, baseline and follow-up images are used as reference interchangeably. Notably, available longitudinal registration pipelines (> 2 timepoints) have limitations in some way or another; for instance, FreeSurfer is restricted to using rigid transforms. Other existing methods, such as NiftyReg and ANTs, can be used for groupwise registration by alternating between mean template computation and pairwise registration to such a template. However, registering each timepoint independently to the template results in jagged longitudinal trajectories. As an example of this sort, the iterative approach presented in Aubert-Broche et al. (2013) is initialised with the standard ICBM152 template.

Geodesic regression approaches using large deformation diffeomorphic metric mapping (LDDMM) have also been explored in longitudinal contexts. A fast learning-based model was proposed by Ding et al. (2019) to predict subject’s momentum from pairwise registrations using the baseline image as a reference template. The work summarised in Bône et al. (2018) iteratively constructs a mean subject’s shape and predicts its trajectory across time. Differently to our approach, they define a statistical model on image intensities, which requires initialising the subject-specific template. In our case, we define a statistical model directly on the image deformations avoiding the computation of the initial template. A similar work to ours, that uses stationary velocity fields (SVFs), is presented in Hadj-Hamou et al. (2016). Their method adopt the baseline image as subject-specific template. Besides, they use a classical registration (non-learning based) algorithm (Lorenzi et al., 2013) which is typically more computationally demanding than learning-based methods. Unlike our work, the computed trajectories lie on MNI space instead of subject space and they do not provide time-consistent segmentations. In Agier et al. (2020), the authors used the same graph structure as our observational graph in the context of groupwise registration of multiple subjects. This structure is very time and memory consuming as it needs pairwise registration of all available timepoints using slow classical registration algorithms. To reduce computational demands, they scale down image dimensionality by working on image keypoints. In our framework we benefit from learning based registration algorithms to deal with the large computational demands of longitudinal registration.

The contributions of this work are threefold:

- First, we present a theoretical framework for longitudinal registration of MRI scans that is explicitly smooth along time, unbiased to any timepoint, and compatible with any number of timepoints and follow up spacing. This method is based on Lie algebra parameterisation of spatial transforms.
- Second, we apply the framework to find subject-specific MRI trajectories that are continuous across time as well as a subject-specific template. For this purpose, we describe two different models of spatial transforms that are applied sequentially: a rigid transform and a non-linear diffeomorphism based on stationary velocity fields.
- Third, we use subject-specific longitudinal deformations to compute time consistent segmentations for all timepoints from initial cross-sectional segmentations using a label fusion approach.

The rest of this manuscript is organised as follows: in Section 2, we describe the “Unbiased and Smooth Longitudinal Registration” (USLR) framework and thoroughly discuss the probabilistic model, the benefits of using Lie algebra parameterisations, the inference method, and the algorithms used for rigid and non-linear registration. In Section 3, we present two methods that build on USLR to compute (*i*) a single stationary subject-specific trajectory and (*ii*) a time-consistent segmentation. In Section 4, we validate and demonstrate the benefits of the USLR framework, illustrated in a use-case group study. Finally, Section 5 summarises the main advantages of USLR as well as discusses the limitations and future directions of this work.

## 2. USLR framework

Our USLR algorithm builds on a probabilistic model of joint diffeomorphic deformations that we first presented in the context of 3D histology reconstruction (Casamitjana et al., 2022). Here, we extend this methodology to impose smoothness and consistency constraints on longitudinal deformations connecting the MRI scans of a subject at different time points. The USLR framework can be applied indistinctly to linear and non-linear transforms as long as the latter are parameterised using stationary velocity fields (SVFs) — thus being compatible with multiple registration algorithms.

In longitudinal modelling, each subject typically undergoes a two step registration process: first, using rigid transforms to model global differences in the orientation (rotation) and position (translation) between observations of the same subject; and second, using non-linear transforms to model local changes in brain tissue, such as changes related to ageing or neurodegeneration. Thus, we present the specificities of the USLR framework particularised with rigid (*USLR-rigid*) and non-linear diffeomorphisms (*USLR-diff*) transforms. In practice, for each subject, we apply the USLR framework in a two-stage process: USLR-rigid is used to bring all subjects in the same shared subject-specific space and USLR-diff is used to measure specific longitudinal changes. We note that the final output is computed using a single interpolation step by concatenating the spatial transforms of both stages. In the rest of this section, we describe the USLR probabilistic model and the parameterisation of rigid and non-linear transforms in the log-space that is required by the USLR inference algorithm — which is subsequently presented.

### 2.1. Preliminaries: graph representation

**Fig. 1.**
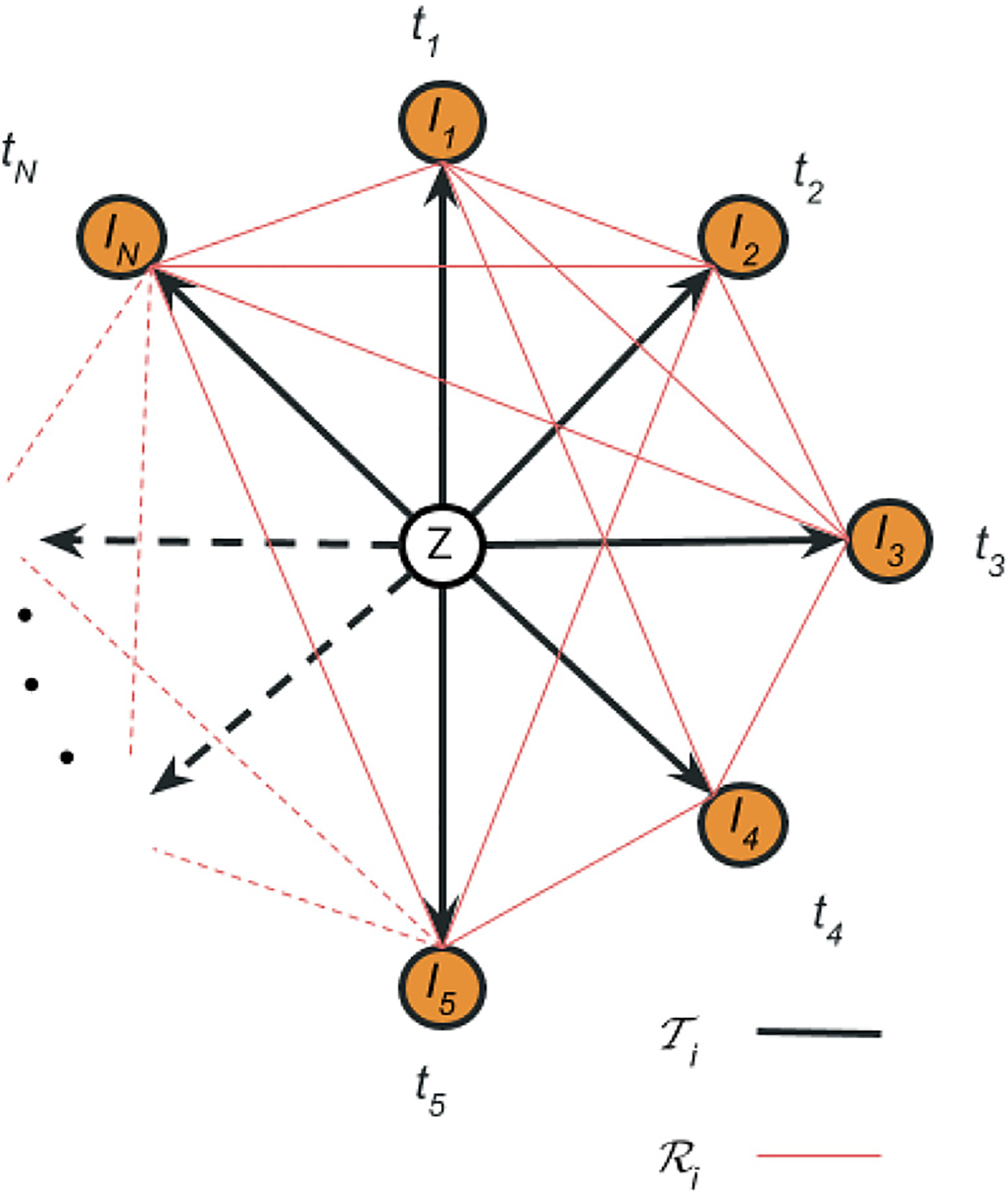
The graph structure 𝒢, where all timepoints “orbit” around the unobserved template. In black, our choice of spanning tree of the graph, where all timepoints are connected through the template. The direction of the associated deformation fields is from the template to the timepoints, as indicated by the arrows In red, we draw the observational graph describing the dense pairwise “noisy” registrations of all timepoints. The direction of this transforms is arbitrary for each subject and known throughout the pipeline. The template is depicted at the centre of masses from all timepoints, ignoring the notion of time between graph nodes. The distance between timepoints and the template and between the template themselves is kept equal to represent the unbiasedness of the method, which treats all timepoints equally.

Let us consider a given subject with a set of N longitudinal MRI scans denoted as $I_{1},I_{2},\ldots ,I_{N}$ acquired at time points $t_{1},t_{2},\ldots ,t_{N}$, respectively. There is no assumption on the spacing between time points (which does not need to be uniform) nor the total number of timepoints. We further represent the N images as vertices of a graph 𝒢, which also contains an additional vertex in the centre — corresponding to a latent subject-specific template (Fig. 1). The vertices corresponding to timepoints are all connected to the centre, creating a spanning tree with N edges associated with a set of N latent transforms ${\left\{{𝒯}_{n}\right\}}_{n=1,\ldots ,N}$ from the template to each image, inducing directionality on the graph 𝒢. These latent transforms need to be invertible such that any pair of timepoints are uniquely related by the composition of two transforms along the edge that connects them: the inverted transform from the first time point to the template and the transform from the template to the second timepoint.

We also consider a set of K=N×(N-1)/2 observed transforms (“registrations”) ${\left\{{ℛ}_{k}\right\}}_{k=1,\ldots ,K}$ between pairs of images of the graph 𝒢. These transforms (shown in red in Fig. 1) are computed with a registration algorithm, such that each $\left\{{ℛ}_{k}\right\}$ can be seen as a noisy version of the composition of two of the latent transforms $\left\{{𝒯}_{n}\right\}$ (which define the “true” underlying deformation) – one of them inverted. Specifically, the registration ${ℛ}_{k}$ between $I_{n}$ (reference) and $I_{n^{\prime }}$ (target) images is simply

(1) $${ℛ}_{k}={𝒯}_{n^{\prime }}\circ {𝒯}_{n}^{-1}\circ {ℰ}_{k},$$

where ${ℰ}_{k}$ represents the registration error (“noise”). Despite the elevated number of registrations, it still remains comparable than the number of registrations in standard iterative algorithms for the typical number of follow-up acquisitions present in clinical studies. For example, USLR computes a fewer number of registrations than ANTs (4 iterations, Avants et al. (2010)) for up to N=9 timepoints, i.e.

$$K_{\text{ANTS}}=4N\geq \frac{N\times (N-1)}{2}=K_{\text{USLR}},\;\forall N=1,\ldots ,9.$$

We note that any other graph configuration (N≤K≤N×(N-1)/2) could be used.

### 2.2. Probabilistic modelling

Similarly to Allassonnière et al. (2007), we assume that, for a given subject, any MRI scan across time is the realisation of a random process that randomly deforms the latent subject-specific template and adds noise to the resulting image:

(2) $$I_{n}\left(x\right)=Z\left(T_{n}^{-1}\left(x\right)\right)+\epsilon \left(x\right),$$

where Z represents the latent (hidden) template, x∈Ω is the spatial location within the image domain Ω, and ϵ(x) is the random noise on the image intensities, whose distribution is arbitrary.

For the latent transforms $\left\{{𝒯}_{n}\right\}$ and the observed registrations $\left\{{ℛ}_{k}\right\}$, we assume a probabilistic model such that we can use Bayesian inference to find a set of transforms that allows us to compute a smooth trajectory across time. The probabilistic model relies on the assumption that the observed registrations are conditionally independent, given the latent transforms, i.e., $p\left(\left\{{ℛ}_{k}\right\}\right)=p\left(\left\{{𝒯}_{n}\right\}\right)\prod_{k}p\left({ℛ}_{k}|\left\{{𝒯}_{n}\right\}\right)$. In general, the likelihood of each registration ${ℛ}_{k}$ is parameterised by a set of parameters $\theta_{k}$ that shape the probabilistic function. In our case, $\theta_{k}$ are the scaling parameters of a Laplace distribution and we consider identical likelihood distributions for all ${ℛ}_{k}$ given $\left\{{𝒯}_{n}\right\}$ (i.e., $\theta_{k}=\theta$), as will be further explained in the following sections. Under these assumptions, the joint probability distribution of the latent transforms, the observed registrations and the likelihood parameters is:

(3) $$p\left(\left\{{𝒯}_{n}\right\},\left\{{ℛ}_{k}\right\},\theta \right)=p\left(\left\{{𝒯}_{n}\right\}\right)p\left(\theta \right)\prod_{k=1}^{K}p\left({ℛ}_{k}|\left\{{𝒯}_{n}\right\},\theta \right).$$

In practice, the term $\text{log}\;p\left(\left\{{𝒯}_{n}\right\}\right)$ can be seen as a regulariser on the latent transforms. This is a bit tangential to typical spatial smoothness constraints in registration problems and we use this regularisation term to impose a constraint on the landscape of possible solutions, as will be further explained through the manuscript.

### 2.3. Simplification with Lie algebra

The likelihood model (Eq.3) is greatly simplified using deformation models that can be parameterised in the Lie algebra space, including linear and non-linear transforms.

Let us define $\left\{R_{k}\right\}$ and $\left\{T_{n}\right\}$ as the log-domain parameterisations whose exponential maps result in the corresponding transformations ${ℛ}_{k}=\text{exp}\;\left[R_{k}\right]$ and ${𝒯}_{n}=\text{exp}\;\left[T_{n}\right]$. The exponential map computation depends on the choice of the deformation model (rigid or non-linear; see Section 2.4 below). Two relevant properties of Lie algebra are specially useful in this framework. First, the inverse transform is exactly equivalent to its negation in the log-space domain:

$${𝒯}_{n}^{-1}=\text{exp}\;\left[-T_{n}\right].$$

Second, the composition of transforms can be approximated by the addition after truncating the Baker–Campbell–Hausdorff series at its first term (Vercauteren et al., 2008):

$${𝒯}_{n}\circ {𝒯}_{n^{\prime }}\approx \text{exp}\;\left[T_{n}+T_{n^{\prime }}\right];$$

These two properties enable us to linearise the probabilistic model in (1) by simply computing the log-space parameterisation:

(4) R=WT+ζ,

where $R\in R^{\text{Kx}|\Omega |}$ and $T\in R^{\text{Nx}|\Omega |}$ contain all the pairwise and latent deformation log-domain parameters of the entire graph and $W\in R^{\text{KxN}}$ is the sparse matrix that encodes the path that any ℛ traverses through the spanning tree (i.e., $\left\{{𝒯}_{n}\right\}$). Hence, for a given spatial transform ${ℛ}_{k}$ between $I_{n}$ (reference) and $I_{n^{\prime }}$ (target) images, the kth row of W is non-zero at entries $W_{kn}=-1$ and $W_{kn^{\prime }}=1$. The ζ is the registration residual in the log-domain. We also assume conditional independence across spatial location, x, and coordinates j=1,…,dim(Ω)=3. These assumptions do not risk the smoothness of the resulting trajectories $\left\{{𝒯}_{n}\right\}$ since they result from a non-linear “average” of smooth observations $\left\{{ℛ}_{k}\right\}$ (Eq. (4)–(6)). As a result, the likelihood function can be written in terms of the log-space parameterisations as

(5) $$p\left({ℛ}_{k}|\left\{{𝒯}_{n}\right\},\theta \right)=p\left(R_{k}|\left\{T_{n}\right\},\theta \right)=\prod_{j=1}^{3}\prod_{x\in \Omega }p\left(R_{k}^{j}(x)|\left\{T_{n}^{j}(x)\right\},\theta \right),$$

for which we use the Laplace distribution

(6) $$R_{k}^{j}\left(x\right)\sim \text{Laplace}\;\left(WT^{j}\left(x\right),b_{T}\right),$$

where the j index refers to the jth dimension of the Lie algebra parameterisations, and $b_{T}$ is the scale of the Laplace distribution and considered the same for all observations, spatial locations and coordinates. The Laplace distribution has the advantage of being robust against registration errors, as shown in Casamitjana et al. (2022). Moreover, it better models the long tails found in practice than the Gaussian distribution (see Section 3 in the supplementary material).

Similar to Wu et al. (2012), we limit the “global drift” across timepoints to estimate a subject space that lies on the centre of masses of all timepoints. Hence, as the prior distribution, we assume that the composition of the latent transforms (approximated by their sum in the log-domain) follows a Laplace distribution centred at zero:

(7) $$\left(\sum_{n=1}^{N}T_{n}^{j}(x)\right)\sim \text{Laplace}\;\left(0,b_{Z}\right),$$

where $b_{Z}$ is the scale of the Laplace distribution, assumed constant for all spatial locations and coordinates. In practice, $b_{Z}$ is large, as the goal of this prior is make the solution unambiguous and centre it at zero. We note that the Laplacian model is used to model registration residual and not for regularising the smoothness of the deformation field, as typically used in registration frameworks. The model parameters are thus $\theta =\left\{b_{T},b_{Z}\right\}$.

### 2.4. Model instantiation

We present two deformation models that we apply sequentially: first, a rigid transformation, that creates a shared space Ω on which the template node is defined, and then a non-linear transformation, which assumes that all timepoints are resampled on Ω. The final latent transforms are computed as the composition of the rigid and non-linear components.

#### 2.4.1. Rigid transforms

Herein this section, let $\left\{{ℛ}_{k}\right\}$ and $\left\{{𝒯}_{n}\right\}$ be the rigid transforms represented by 4 × 4 matrices as:

$$\left(\begin{array}{cc} U & t\\ 0_{1\times 3} & 1 \end{array}\right),$$

where U is the 3 × 3 rotation matrix and t the 3–dim translation vector. The group of rigid transformations in $R^{3}$ constitute the special Euclidean group 𝒮ℰ(3) and can be parameterised in the log-space domain using a 6-dimensional vector $(q,d{)}^{⊤}$ with two 3-dimensional components: $q\in R^{3}$ and $d\in R^{3}$, that determine the rotation and translation, respectively (Blanco-Claraco, 2021). Thus, the Lie algebra parameterisations of the transforms are $R_{k}={\left(q\left(R_{k}\right),d\left(R_{k}\right)\right)}^{⊤}$ and $T_{n}={\left(q\left(T_{n}\right),d\left(T_{n}\right)\right)}^{⊤}$. Note that we drop the spatial dimension x as the parameters are independent of the location.

To compute the log-space parameters, we use the following expressions (Blanco-Claraco, 2021):

$$q=\frac{\phi }{2\;\text{sin}\;\phi }{\left(U_{32}-U_{23},U_{13}-U_{31},U_{21}-U_{12}\right)}^{⊤},$$

(8) $$d=P^{-1}t,$$

where $U_{ij}$ is the matrix value corresponding to the ith row and jth column of matrix U, and (ϕ, P) can be computed as:

$$\phi =\text{arccos}\left(\frac{1}{2}(\text{tr}(U)-1)\right),$$

$$P^{-1}=I_{3}+0.5Q+\frac{\left(1-\frac{\phi \;\text{cos}\;\phi /2}{2\;\text{sin}\;\phi /2}\right)}{\phi^{2}}Q^{2},$$

where

$$Q=\left(\begin{array}{ccc} 0 & -q_{z} & q_{y}\\ q_{z} & 0 & -q_{x}\\ -q_{y} & q_{x} & 0 \end{array}\right)\;\text{and}\;q={\left[q_{x},q_{y},q_{z}\right]}^{⊤}.$$

To compute the exponential maps, we use the closed form expressions for the Lie group parameters, U and t, in Blanco-Claraco (2021):

(9) $$U=I_{3}+\frac{\text{sin}\;\phi }{\phi }Q+\frac{\left(1-\text{cos}\;\phi \right)}{\phi^{2}}Q^{2},$$

(10) $$t=\left(I_{3}+\frac{\left(1-\text{cos}\;\phi \right)}{\phi^{2}}Q+\frac{\phi -\text{sin}\;\phi }{\phi^{3}}Q^{3}\right)d,$$

where ϕ=|q|. We note that ϕ refers to the angle of rotation to a fixed Cartesian coordinate system is known given a rotation matrix U or its Lie algebra parameterisation q (Blanco-Claraco, 2021).

#### 2.4.2. Non-rigid diffeomorphisms

Here, we assume that all images are rigidly aligned and resampled onto the same (discrete) spatial domain Ω, which we refer to as *subject space*. In practice, this subject space is an arbitrarily defined 1 mm isotropic grid (further details in Section 2.6). In this scenario, non-linear deformations are relatively small compared to inter-subject deformations. Hence, the simplification from Section 2.3 is a good approximation of field composition as empirically shown in the supplementary material (Section 3), where we provide a comparison of the error incurred by such approximation. Therefore, we parameterise a class of non-linear diffeomorphisms using the Lie group of stationary velocity fields (SVFs, (Arsigny et al., 2006)). Let $\left\{R_{k}(x)\right\}$ and $\left\{T_{n}(x)\right\}$ be the SVF infinitesimal generators in the log-space whose integration results in the corresponding diffeomorphisms ${ℛ}_{k}=\text{exp}\;\left[R_{k}\right]$ and ${𝒯}_{k}=\text{exp}\;\left[T_{k}\right]$. The scaling-and-squaring approach is used for fast computation of these exponentials (Arsigny et al., 2006).

### 2.5. Inference algorithm

Following the formulation in Section 2.2, we use Bayesian inference to compute the most likely set of N transforms $\left\{{𝒯}_{n}\right\}$ that generate the pairwise image registrations $\left\{{ℛ}_{k}\right\}$. In a fully Bayesian formulation, the problem of finding the most likely latent transforms requires marginalisation over the parameters we are not seeking to optimise, in this case $\theta =\left\{b_{T},b_{Z}\right\}$. However, the relationship between these hyperparameters is assumed to be known, yielding the following optimisation function:

(11) $$\left\{{\overset{ˆ}{T}}_{n}\right\}=\underset{\left\{T_{n}\right\},\theta }{\text{argmax}}\;p\left(\left\{T_{n}\right\},\theta ,\left\{R_{k}\right\}\right)\;=\underset{\left\{T_{n}\right\},\theta }{\text{argmax}}\;p\left(\left\{T_{n}\right\}\right)\prod_{k=1}^{K}\prod_{j=1}^{3}\prod_{x\in \Omega }p\left(R_{k}^{j}(x)|\left\{T_{n}^{j}(x)\right\},\theta \right)\;=\underset{\left\{T_{n}\right\},\theta }{\text{argmax}}\;\text{log}\;p\left(\left\{T_{n}\right\}\right)+\sum_{k=1}^{K}\sum_{j=1}^{3}\sum_{x\in \Omega }\text{log}\;p\left(R_{k}^{j}(x)|\left\{T_{n}^{j}(x)\right\},\theta \right).$$

Substituting the Laplacian likelihood and prior from Eqs. (6) and (7) into Eq. (11), we obtain the following objective function:

(12) $${𝒪}_{\ell_{1}}=-2\times 3(K+1)|\Omega |\text{log}\left(2b_{T}\right)-2\times 3|\Omega |\text{log}\left(2b_{Z}\right)\;-\frac{1}{b_{Z}}\sum_{j=1}^{3}\sum_{x\in \Omega }\left|\sum_{n=1}^{N}T_{n}^{j}(x)\right|\;-\frac{1}{b_{T}}\sum_{j=1}^{3}\sum_{k=1}^{K}\sum_{x\in \Omega }\left|R_{k}^{j}\left(x\right)-\sum_{n=1}^{N}W_{kn}T_{n}^{j}\left(x\right)\right|.$$

To keep it general, we retain the dependency on the spatial coordinates and location needed for the non-linear model; note that we would drop it for the rigid case.

Rearranging terms and switching signs, the cost function to minimise results as follows:

(13) $$C_{\ell_{1}}(T(x))=\frac{b_{T}}{b_{Z}}\sum_{j=1}^{3}\sum_{x\in \Omega }\left|\sum_{n=1}^{N}T_{n}^{j}(x)\right|\;+\sum_{j=1}^{3}\sum_{k=1}^{K}\sum_{x\in \Omega }\left|R_{k}^{j}(x)-\sum_{n=1}^{N}W_{kn}T_{n}^{j}(x)\right|,$$

which can be solved one spatial location x and coordinate at a time. Thus, we independently solve

(14) $$C_{\ell_{1}}\left(T^{j}(x)\right)=\frac{b_{T}}{b_{Z}}\left|\sum_{n=1}^{N}T_{n}^{j}(x)\right|+\sum_{k=1}^{K}\left|R_{k}^{j}(x)-\sum_{n=1}^{N}W_{kn},T_{n}^{j}(x)\right|,$$

at every x. After visual inspection of the resulting velocity fields, we empirically set the hyperparameter relationship to 1, i.e., $b_{T}/b_{Z}=1$, as a trade-off between smoothness and accuracy. Note that in the case of rigid transforms, we solve all parameters at once and as they are the same for every spatial location. In the case of non-linear diffeomorphisms, we solve Eq. (14) at control points set at 1/8 the original image resolution, which represents a good compromise between local smoothness and independence between points far apart.

The minimisation of Eq. (14) can be rewritten as a linear program in standard and solved using well-established linear programming algorithms, such as HiGHS (Huangfu and Hall, 2018) (used here) or interior-point methods (Karmarkar, 1984; Andersen and Andersen, 2000). A more detailed description of the linear program can be found in the supplementary material (Sec.2)

### 2.6. Subject-specific template

The template space is defined on a 1 mm^3^ isotropic grid Ω. After the first step of the pipeline (USLR-rig), we find the subject space that includes the rigidly registered images from all timepoints. This space is characterised by a cuboid and an affine matrix mapping voxel and physical coordinates. The cuboid size is computed as the minimal size that covers the images from all timepoints plus a margin of 5 voxels in each direction; the affine matrix is defined as a RAS oriented diagonal matrix plus a translation to centre the image at the origin (0, 0, 0) mm in world coordinates. This space is where the subject-specific template is defined.

The subject specific template Z(x) is built after the second step of the framework (USLR-diff) and consists of the intensity image, $Z_{I}(x)$, a brain mask, $Z_{M}(x)$ and a segmentation map, $Z_{S}(x)$, if available. In our case, we use SynthSeg (Billot et al., 2023b) to produce brain segmentation maps for all images (which are thresholded to yield brain masks). To build such a template, we first align all timepoints (intensities, masks, and one-hot encoded segmentation maps) to the template space using a composition of the rigid and non-linear deformation fields. Trilinear interpolation from original images is used for resampling. To calculate the template intensity image we take the median value of all resampled images as the optimal solution for the Laplacian noise defined in Eq. (2). For template brain mask, we employ the mean value of all aligned timepoints masks as a measure of brain tissue probability. And segmentation maps are computed as the most likely value after taking the mean on the deformed one-hot encodings of the label volumes.

## 3. USLR application examples

USLR outcomes could serve a variety of applications and downstream tasks. Here, we present 2 different applications: first, an estimation of a single stationary velocity field that characterises the time course of the subject and that could be used, for example, in tensor-based population analyses; and second, a label fusion approach for image segmentation with longitudinal constraints. Note that no assumption on the original image resolution nor the contrast is made. Any resampling made throughout the pipeline is always computed from the original images (i.e., concatenating transforms) to avoid resampling biases due to smoothing.

### 3.1. Stationary subject-specific longitudinal trajectory

The composition of the estimated non-linear latent transforms yields a smooth subject-specific longitudinal trajectory that is varying across time and not defined outside the followup period of each subject. The purpose of this step is to estimate a single stationary longitudinal trajectory, $\overset{ˆ}{𝒯}$, as the exponential map of a linear fit on the SVF parameterisation of latent transforms, $\overset{ˆ}{T}$:

(15) $$\overset{ˆ}{𝒯}(x,t)=\text{exp}(t\cdot \overset{ˆ}{T}(x)).$$

For that, we use a voxelwise linear model on the SVF maps for each spatial direction and location as follows:

(16) $$T_{n}^{j}(x)=c^{j}(x)+{\overset{ˆ}{T}}^{j}(x)\cdot t_{n},\;\text{with}\;j\in \{x,y,z\}.$$

where $t_{n}$ is the time to baseline of the nth timepoint and c(x) is the constant map that shifts the origin to the template image.

As in Section 2.4.2, we assume independence across coordinates and spatial locations. Despite this fact, we observe that smooth latent deformations $\left\{𝒯(x{)}_{n}\right\}$ lead to a smooth subject-specific longitudinal trajectory $\overset{ˆ}{𝒯}(x,t)$. We emphasise that using a linear model on the SVF maps does not lead to a linear model on the deformations due to the exponential relation, as seen in Eq. (15).

This approximation of the subject-specific trajectory can be used, for example, for linear prediction of future timepoints by deforming the non-linear template at a given time. It constitutes a longitudinal signature of brain anatomical changes for each subject that could be used for population studies, such as tensor- and deformation-based morphometry (TBM/DBM).

### 3.2. Longitudinal segmentation

Capitalising on the N latent transforms (rigid and non-rigid) that connect all images through the spanning tree defined in Section 2.1, we compute a time-consistent segmentation on each timepoint space employing the label fusion strategy in Sabuncu et al. (2010) on the deformed cross-sectional labelmaps. These initial segmentations could be computed using any manual, automatic and semi-automatic method or a combination of those, as long as all timepoints follow the same delineation protocol. In our case, we use SynthSeg as a cross-sectional segmentation tool for labelmap initialisation (Billot et al., 2023b). Original T1-w images are corrected for intensity inhomogeneities (Van Leemput et al., 1999), normalised such that the mean value of white matter voxels is 110 and used in the weighted label fusion step (Sabuncu et al., 2010).

In short, given the nth observation defined as reference, we linearly resample both the T1w image and the one-hot encoded labelmaps of the N-1 remaining timepoints $\left(n^{\prime }\neq n\right)$ to the space of the nth timepoint using the computed latent transforms. All prior segmentations (N) are used in the following weighted average label fusion. The weights are proportional to the image likelihood using a Gaussian kernel with a standard deviation of 3 on the intensity differences between the reference and the deformed images. Hence, the nth prior segmentation will always contribute more than the remaining timepoints as $I_{n}-I_{n}=0\;\forall x\;\in \;\Omega_{n}$. The label fusion step can also be seen as the MAP estimate using the image likelihood and prior segmentation maps of all timepoints thus inducing consistency across time.

### 3.3. Subject alignment to MNI

For tensor-based and deformation-based morphometry (TBM/DBM) analyses, we spatially normalise the subject-specific maps to the MNI space. We use SynthMorph to non-linearly register the subject-specific T1-w template to the MNI2009a non-linear template (see Section 1.2 from the supplementary material for the details). To normalise scalar quantities to MNI (e.g., subject-specific template, Jacobian determinant maps), we directly resample the 3D map using the computed deformation field.

## 4. Experiments and results

### 4.1. Data

We use the Minimal Interval Resonance Imaging in Alzheimer’s Disease (MIRIAD) dataset (Malone et al., 2013) to exhaustively show the benefits of using the USLR framework for groupwise analysis in longitudinal settings. The MIRIAD dataset is a structural MRI cohort of 46 Alzheimer’s disease (AD) subjects and 23 elderly healthy ageing adults with multiple available T1w scans collected at different time points. Follow up periods per subject range up to two years and sampling points are unevenly spaced from 2 weeks to one year. Moreover, test-retest scans are available at baseline, and 6 and 38 weeks from baseline. We consider two separate subsets from the MIRIAD study: first, we build a small set using the baseline test and retest images as two different timepoints; and second, we build a larger set consisting of all available sessions for all subjects excluding the re-test images. We process all subjects through our USLR processing pipeline as well as through the longitudinal stream of FreeSurfer for comparison with a standard pipeline widely accepted by the community. The Alzheimer’s disease neuroimaging initiative (ADNI) dataset is used as a large scale and more heterogeneous dataset. The ADNI (adni.loni.usc.edu) was launched in 2003 as a public–private partnership, led by Principal Investigator Michael W. Weiner, MD. The primary goal of ADNI has been to test whether serial magnetic resonance imaging (MRI), positron emission tomography (PET), other biological markers, and clinical and neuropsychological assessment can be combined to measure the progression of mild cognitive impairment (MCI) and early Alzheimer’s disease (AD). We select a subset of 54 healthy control subjects (CN), 73 mild cognitive impairment (sMCI) subjects, 64 Alzheimer’s disease (AD) patients and 78 subjects that converted from MCI to AD (cMCI) during the time span of the study. Diagnostic labels are extracted from the ADNI cohort. The average number of timepoints per subject is 5.48 (±1.75) with follow up periods of 3.5 (±1.67) years.

Furthermore, an Alzheimer’s disease patient from our local dataset is used to better illustrate the benefits of subject-specific analyses. Recruited at the age of 87.5, a total of 11 timepoints across 7.5 years of follow up with its associated T1w scans are readily available and processed through the USLR pipeline.

### 4.2. Unbiased and smooth trajectories

Two of the main properties of the pipeline are the unbiasedness of the template and the smoothness of the resulting trajectories.

**Fig. 2.**
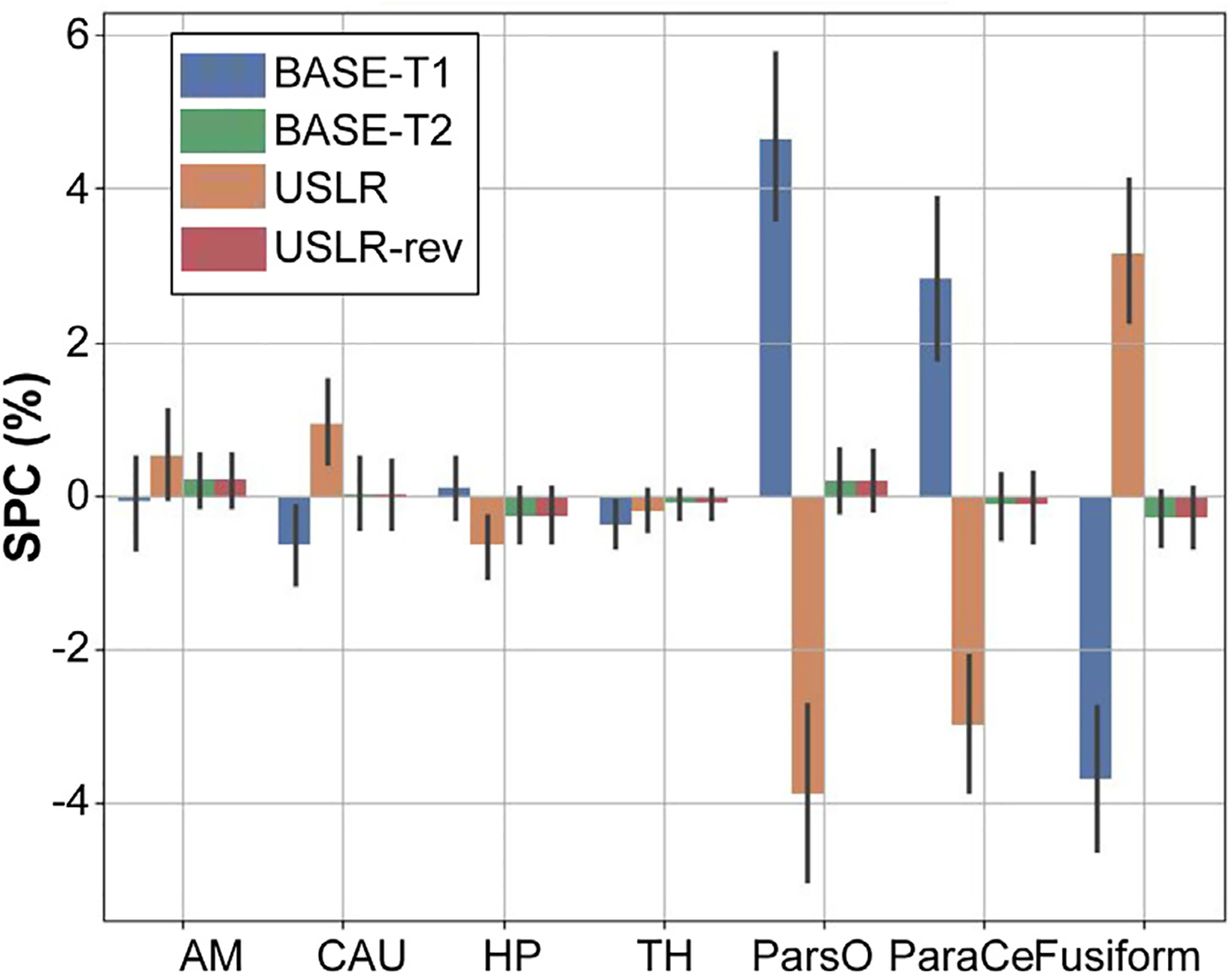
Symmetrised percent change (SPC) for different cortical and subcortical brain regions: amygdala (AM); caudate (CAU); hippocampus (HP); thalamus (TH); pars opercularis (ParsO); paracentral (ParaCe); and fusiform. BASE-T1 (T2) initialises timepoint 2 (1) with the segmentation of timepoint 1 (2). USLR and USLR-rev process timepoints in reversed order.

As argued in Yushkevich et al. (2010) and Reuter et al. (2012), asymmetries in the processing can cause bias in longitudinal processing. We prevent interpolation bias by treating all timepoints equally. Despite the fact that some timepoints will be closer to the template than the others, all timepoints equally contribute to the final output of the pipeline. We note that the final subject-specific template will approximately lie on the centre of masses of all timepoints. To demonstrate the effect of asymmetries, we use the segmentation from one timepoint to initialise the processing of another timepoint on the test-retest MIRIAD subset and compute the symmetrised percent change (SPC), defined as

(17) $$\text{SPC}=100\frac{2\left(V_{2}-V_{1}\right)}{V_{1}+V_{2}},$$

where $V_{i}$ is the volume of a given structure at the ith timepoint. In Fig. 2, we show the SPC for 4 subcortical structures and 3 cortical parcellations when processing test-retest data using timepoint 1 to initialise the segmentation of timepoint 2 (BASE-T1) and viceversa (BASE-T2). We also report the results of the USLR segmentation with timepoints ordered differently (USLR, USLR-rev). Since both timepoints come from the same session, one could expect zero difference between BASE-T1 and BASE-T2 but we see differences in subcortical and cortical regions attributable to the asymmetry. Almost inexistent differences can be seen between USLR and USLR-rev, which is expected due to the construction of the USLR framework.

**Fig. 3.**
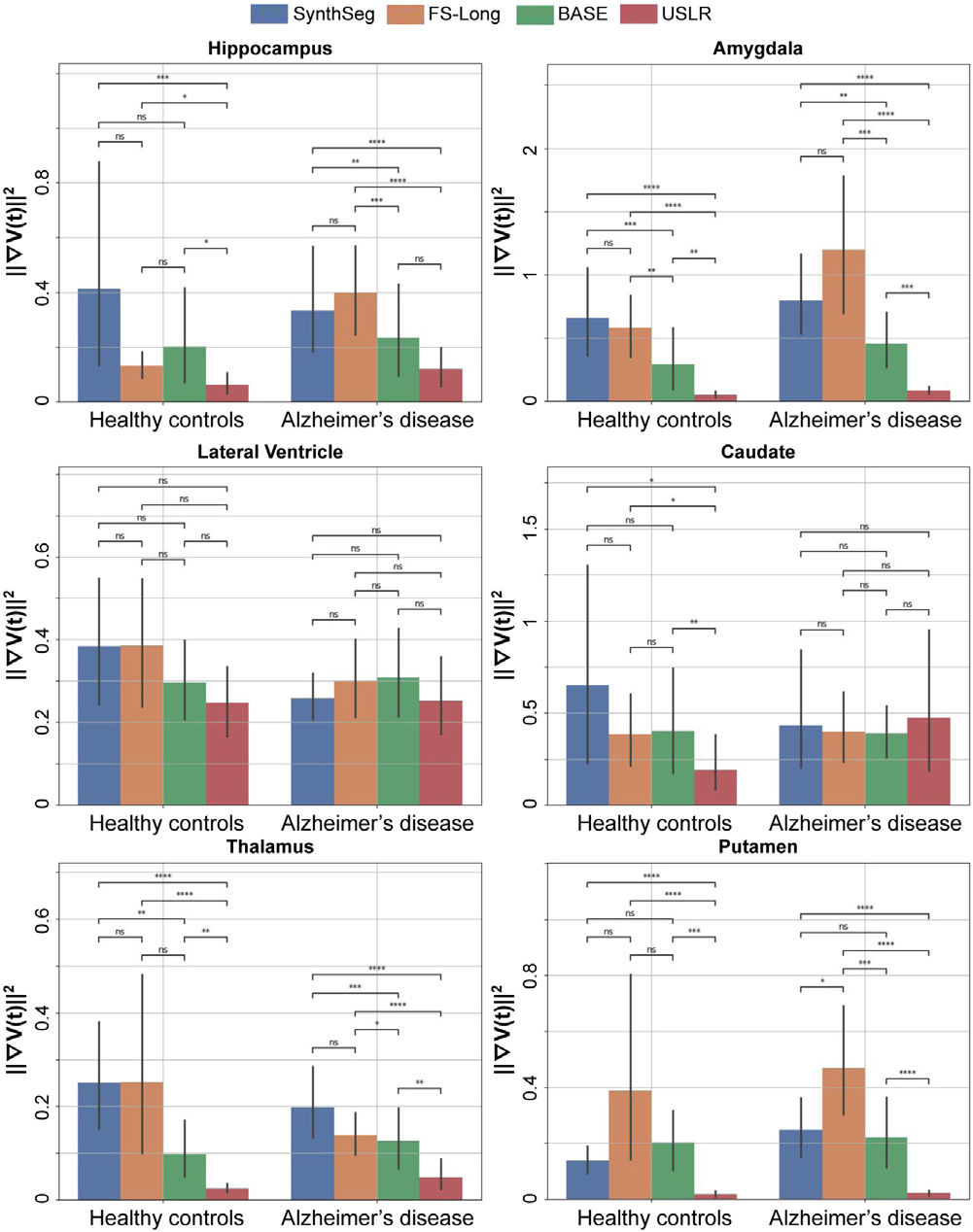
Evaluating trajectory smoothness using Eq. (18) stratified by diagnostic category. We compare four different models: SynthSeg, longitudinal Freesurfer segmentations (FS-Long), longitudinal refinement using the baseline image as template (BASE) and USLR. Significant differences in smoothness are found in a Wilcoxon-rank test between methods for (*) $1\cdot 10^{-2}<p<5\cdot 10^{-2}$, (**) $1\cdot 10^{-3}<p<1\cdot 10^{-2}$, (***) $1\cdot 10^{-4}<p<1\cdot 10^{-3}$ and (****) $p<1\cdot 10^{-4}$ thresholds.

To demonstrate the smoothness of the result, we compute the volumetric trajectories of all subjects using all available timepoints of the MIRIAD dataset, excluding test-retest data. The smoothness of each trajectory over time can be characterised by the $\ell_{2}$-norm of the finite differences between consecutive timepoints,

(18) $$\|\nabla V(t){\|}^{2}=\sum_{n=0}^{N-2}{\left(\frac{V_{n+1}-V_{n}}{t_{n+1}-t_{n}}\right)}^{2}.$$

where $V_{n}$ is the volume of a given structure at the ith timepoint for a given subject. Fig. 3 shows this quantity for different methods: (i) cross-sectional SynthSeg, (ii) longitudinal stream of Freesurfer; (iii) longitudinal modelling using the baseline image as reference template; and (iv) USLR. We compare all four methods using the Wilcoxon-rank test. It can be seen that USLR is significantly smoother than the others for many regions regardless of the diagnostic category (Amygdala,Thalamus, Putamen) and for healthy cognitive ageing in others (Hippocampus, Caudate). We argue that the lateral ventricle is a much simpler structure to segment and all methods perform similarly. Further smoothness analysis could be found in Section 5.2 of the supplementary material.

### 4.3. Subject-specific outcomes

**Fig. 4.**
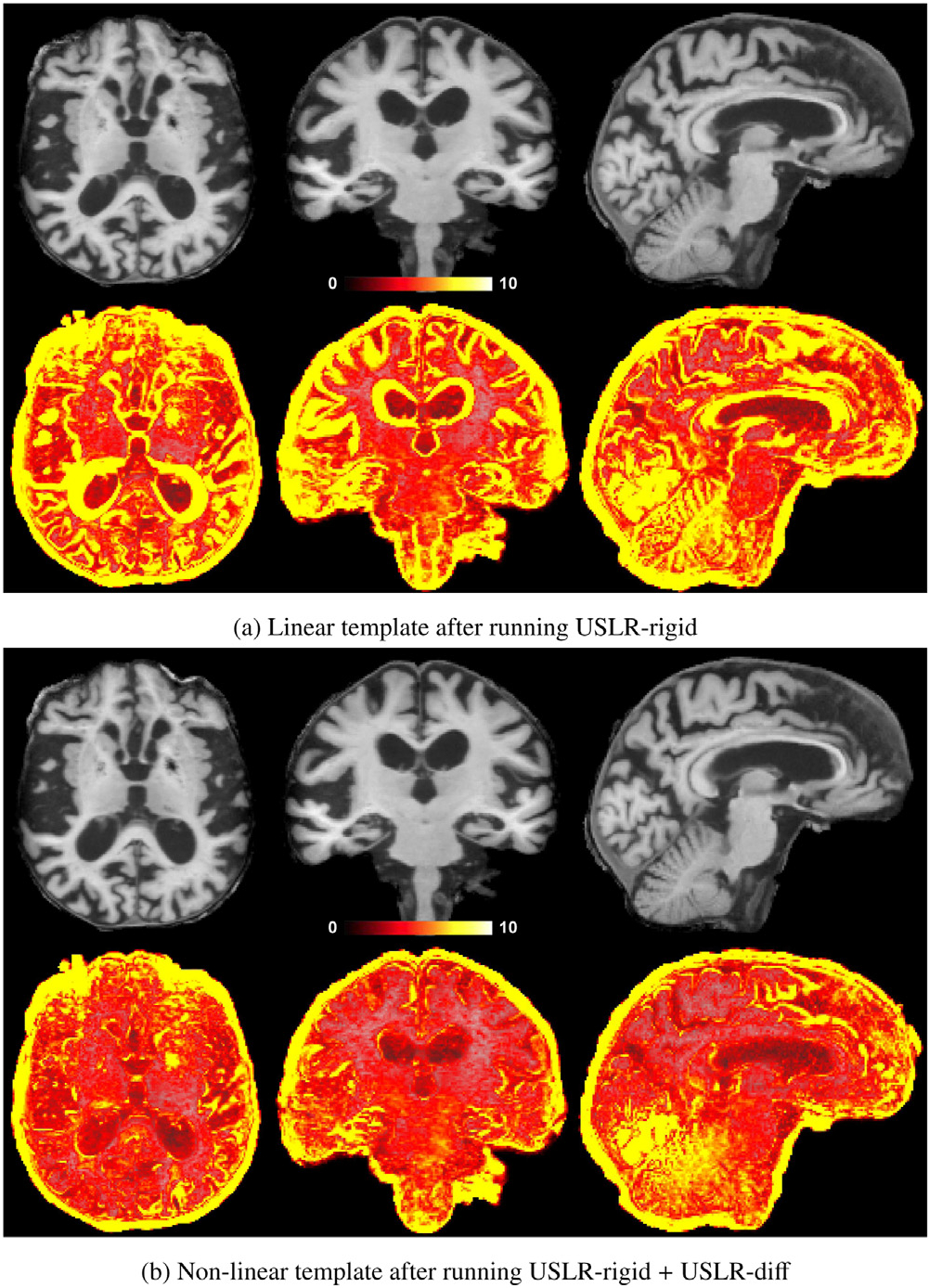
Example of a template of an Alzheimer’s disease subject followed-up for a total of 7.5 years with L=11 scans from a local cohort. On the top row, the mean T1w subject-specific template and on the bottom row, the standard deviation of the intensities between all the resampled and normalised timepoints. The larger variance in the linear template leads to misleading intensity values, such as around the ventricles, where it looks like grey-matter tissue. The non-linear template is sharper and prevents artificial intensity values.

We use the Alzheimer’s disease patient from our local cohort to illustrate the straightforward subject-specific outcomes from the USLR pipeline. On the one hand, the computed smooth longitudinal subject-specific trajectories and the resulting unbiased non-linear template. While the benefits of avoiding bias are obvious and discussed in Reuter et al. (2012), the use of non-linear transforms yield sharper and more realistic templates compared to linear transforms, as shown in Fig. 4. In standard longitudinal processing frameworks, such as Freesurfer, a subject-specific template is built using rigid and/or linear transforms from all timepoints, which we refer to as “linear template”. In our framework, the non-linear stream aims at modelling atrophy and local deformation and adds-up to the initial linear alignment. The resulting better alignment between the template and timepoints reduces the intensity differences among them and avoids artificial intensity values in the image. We refer to this latter subject-specific template as “non-linear template”. We note that the final template is computed by concatenating USLR-rig and USLR-diff transforms and resampling original images to subject space only once.

**Fig. 5.**
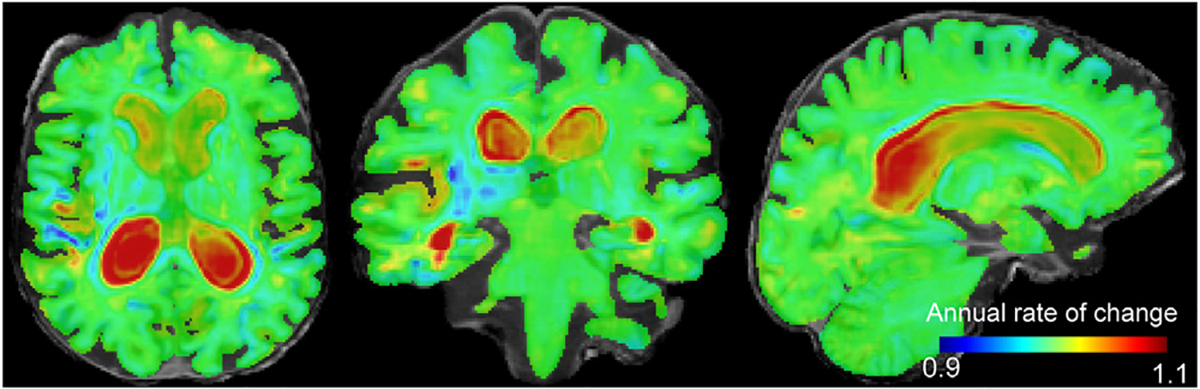
Jacobian determinant of the 1-year subject-specific estimated trajectory of an Alzheimer’s disease patient overlaid on its non-linear template. Hot colours (> 1) indicate expansion and cold colours (< 1) indicate contraction over time. The value of each voxel indicates the annual rate of change.

On the other hand, the subject-specific longitudinal signature of brain change (stationary trajectory), computed as explained in Section 3.1. Such trajectory can be integrated, using the scaling-squaring algorithm (Arsigny et al., 2006), at any given time to quantify the expected volumetric rate of change. In the supplementary material (Section 5.1) we evaluate the goodness of fit of this approximation and compare to other methods in the literature. Using tensor based morphometry (TBM), we compute the Jacobian determinant of the deformation to create 3D structural maps of local atrophy. An example of the rate of change over 1 year of an Alzheimer’s disease patient is presented in Fig. 5, showing the typical expansion around the ventricles and shrinkage of some grey matter regions in the cortex or temporal lobe.

**Fig. 6.**
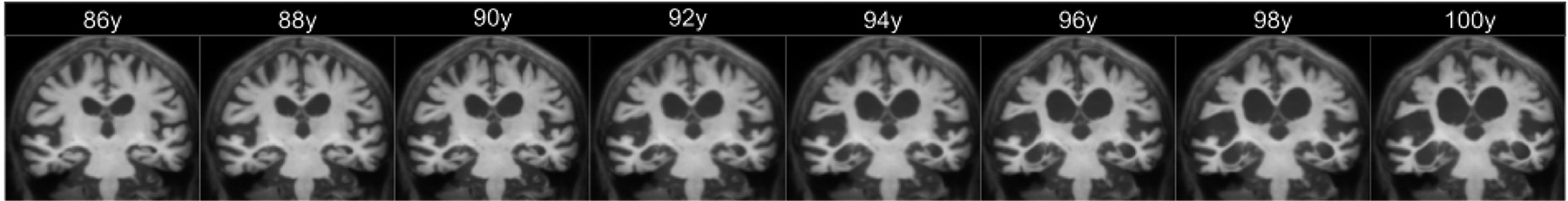
Subject-specific prediction at the voxel level, computed by deforming the non-linear subject-specific template using the estimated SVF trajectories. The age range shown spans from 1.5 years prior the baseline observation to 5 years after the last observation. The real duration of the study ranges from 87.5 to 95 years old.

This stationary trajectory can be used for prediction. Due to the SVF parameterisation, the trajectories could be easily inverted to compute forward and backward deformations. Thus, we could interpolate between observations (e.g. missing timepoints) and extrapolate outside the follow up period (prediction) with no blurring. An example of subject-specific evolution that expands the observed period both prior to the baseline image after the last timepoint is shown in Fig. 6

### 4.4. Population analyses

We now use the computed longitudinal trajectories and segmentations for population studies usind the MIRIAD and ADNI cohorts.

#### 4.4.1. Tensor-based morphometry

**Fig. 7.**
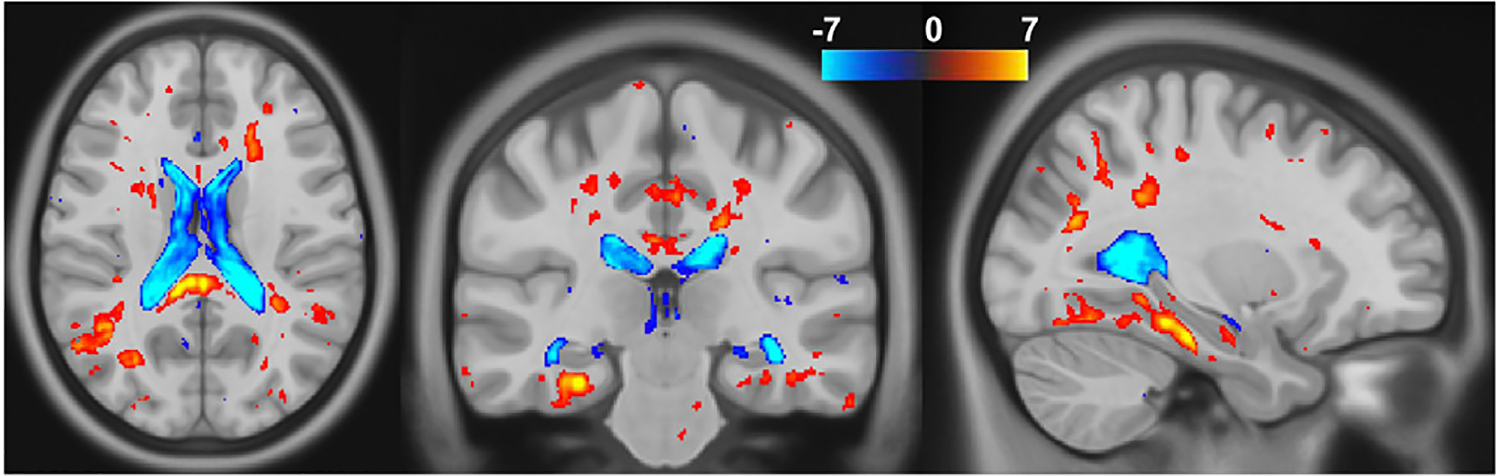
T-test results on the Jacobian determinants between healthy controls and Alzheimer’s disease subjects, computed using USLR. We show the t-values thresholded at p=0.05 corrected for multiple comparisons using FDR.

First, we investigate statistical groupwise differences on the longitudinal trajectories between Alzheimer’s disease subjects and ageing adults without cognitive deficits from the MIRIAD dataset. We compare the Jacobian determinant maps of each group using a voxelwise two-sample t-test. To compute the Jacobian maps we use the stationary subject-specific trajectory introduced in Section 3.1 (i.e., one trajectory per subject) and integrate it over the period of 1 year. We further resample the Jacobians to MNI space as explained in Section 3.3. The null hypothesis is that there is no difference on the rate of change between groups. We correct for multiple comparisons using a false discovery rate (FDR) strategy with a corrected p-value threshold at 0.05 for statistical significance. In Fig. 7 we show the thresholded t-value maps overlaid on the MNI2009a template. We see significant positive and negative differences that can be interpreted as volumetric differences in typical AD-related regions. In particular, in a one-year period, we see increased volume in the ventricular areas (Nestor et al., 2008) and reduced volume in the cortical regions such as the entorhinal cortex (Contador et al., 2021) and subcortical regions such as mediodorsal thalamic nuclei (Iglesias et al., 2018), among others. Further comparison to other methods in the literature can be found in the supplementary material (Section 5.3)

#### 4.4.2. Test-retest reliability

**Fig. 8.**
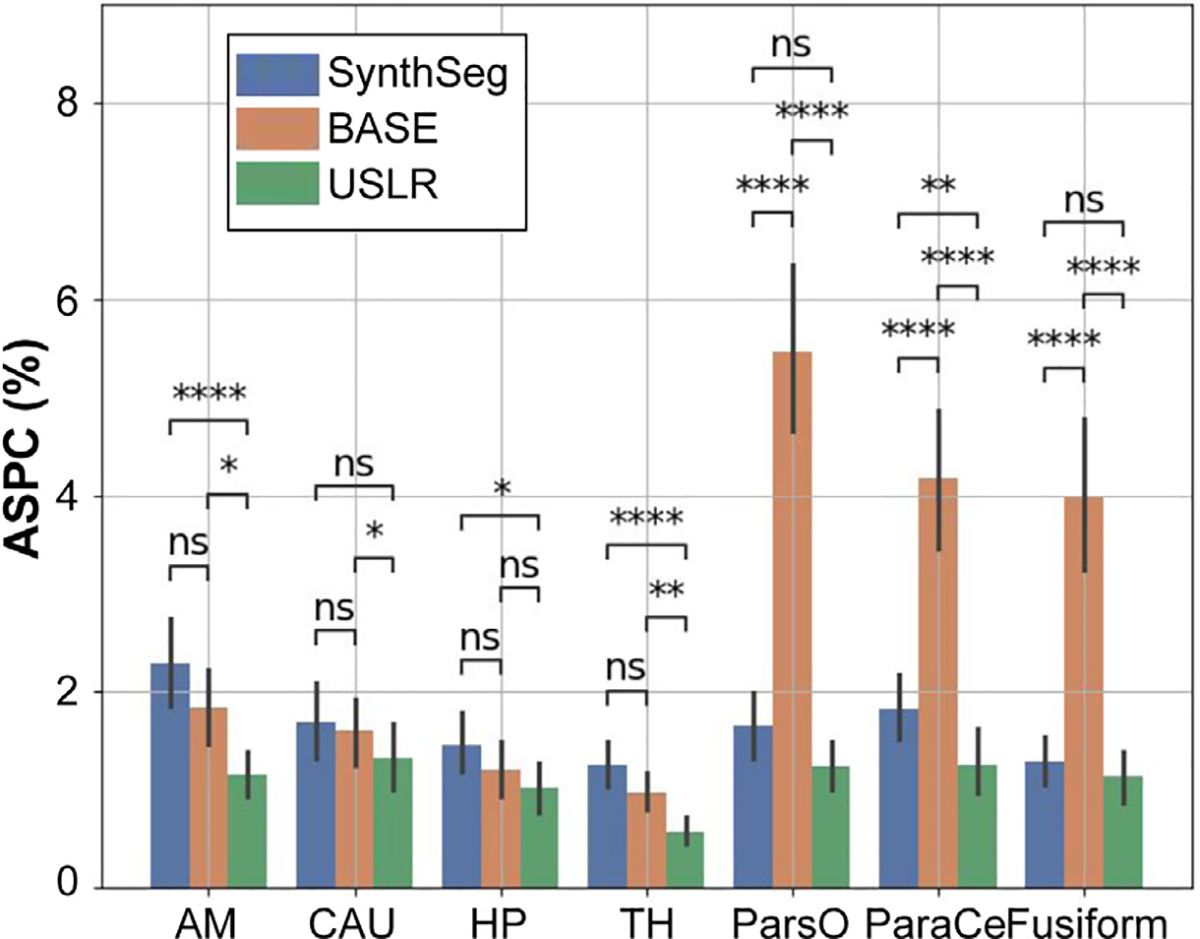
Absolute symmetrised percent change (ASPC) for different cortical and subcortical brain regions: amygdala (AM); caudate (CAU); hippocampus (HP); thalamus (TH); pars opercularis (ParsO); paracentral (ParaCe); and fusiform. Three segmentation methods are compared: (i) SynthSeg, which is the original cross-sectional segmentations; (ii) BASE, which is the longitudinal refinement using one acquisition as reference template; and (iii) USLR. A Wilcoxon-rank test is used for statistical significance with (*) $1\cdot 10^{-2}<p<5\cdot 10^{-2}$, (**) $1\cdot 10^{-3}<p<1\cdot 10^{-2}$, (***) $1\cdot 10^{-4}<p<1\cdot 10^{-3}$ and (****) $p<1\cdot 10^{-4}$ thresholds.

To assess the reliability of the segmentation measurements we measure the within session variability on the MIRIAD test-retest set. We treat the two acquisitions as separate timepoints compute the absolute symmetrised percent change (ASPC),

(19) $$\text{ASPC}=100\frac{2\left|V_{2}-V_{1}\right|}{V_{1}+V_{2}},$$

where $V_{1}$ and $V_{2}$ are the test and retest volumetric observations of a given brain region. Fig. 8 compares the original cross-sectional segmentations a longitudinal approach using one acquisition as reference template and USLR. Our unbiased longitudinal process consistently improve the original SynthSeg across multiple brain regions, improving the within session reliability and reducing potential undesired confounds.

### 4.5. Sensitivity analyses

**Fig. 9.**
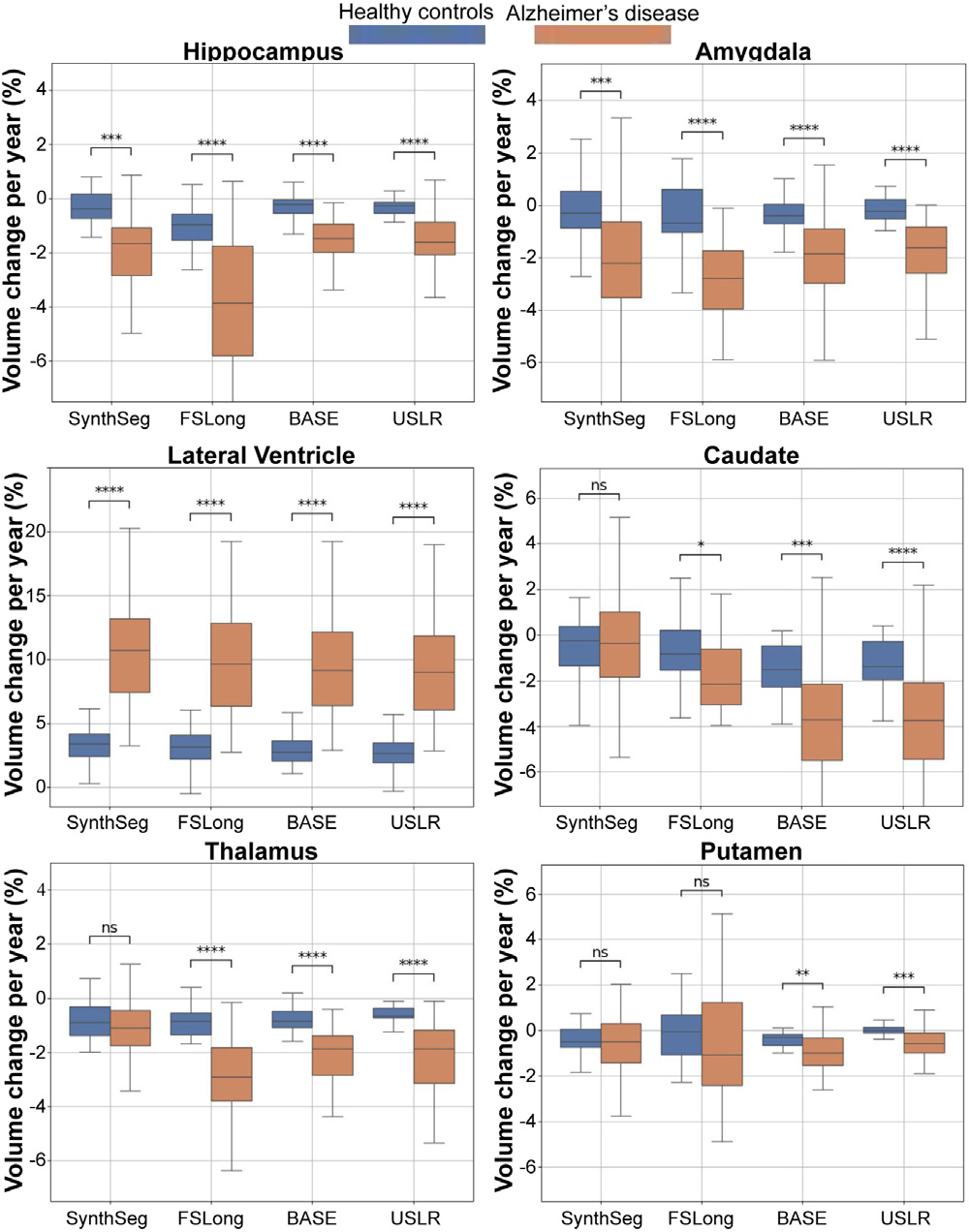
Sensitivity analysis computing the trajectory’s slope of 6 different ROI volumes per subject. In each figure, we compare three different segmentation methods. From left to right: cross-sectional SynthSeg, the longitudinal stream of Freesurfer, a longitudinal refinement using the baseline image as template and our USLR framework. Cognitively normal subjects are grouped in blue while AD subjects in dark orange. Significant differences in mean slopes are found in a Wilcoxon-rank test between groups for (*) $1\cdot 10^{-2}<p<5\cdot 10^{-2}$, (**) $1\cdot 10^{-3}<p<1\cdot 10^{-2}$, (***) $1\cdot 10^{-4}<p<1\cdot 10^{-3}$ and (****) $p<1\cdot 10^{-4}$ thresholds.

Nonetheless, a very naive algorithm that outputs always the mean value between timepoints would have ideal *ASPC* despite not being able to capture changes between acquisitions. Thus, we also study the sensitivity of the framework in detecting changes between group’s trajectories on the entire MIRIAD dataset. We compare USLR with the original SynthSeg and two longitudinal processing approaches, namely, Freesurfer longitudinal segmentations and a longitudinal refinement using the baseline image as template. In Fig. 9, we show the yearly rate of change with respect the baseline volume for different subcortical regions computed as the mean value between hemispheres. For a more robust estimate, we use the prediction of the linear fit for each subject at t=0 as the baseline volume instead of the value of its first timepoint. Statistical significance is computed using the Wilcoxon-rank test between groups. Clearly, the reduced inter-subject variability of longitudinal processing improves the discrimination power between groups, sometimes at the very mild cost of reduced volumetric change per year. Moreover, we also see less variability between atrophy rates from USLR compared to Freesurfer, specially for the control group.

#### 4.5.1. Power analysis

These results indicate the suitability of the framework on detecting subtle differences in the rate of change between diagnostic categories which may impact the power of the study or the required sample size. Following Diggle (2002) and Reuter et al. (2012) nomenclature, the minimum sample size of a longitudinal study could be computed as

(20) $$m=\frac{2{\left(z_{\alpha }+z_{1-P}\right)}^{2}\sigma^{2}(1-\rho )}{Ns_{x}^{2}d^{2}},$$

where m is the sample size per each group, σ is the unexplained standard deviation of the observations, ρ is the correlation of repeated observations, d is the target effect size, N is the number of timepoints per subject, $s_{x}$ the within-subject variance of the variable of interest (in this case, the time between acquisitions and constant across subjects), P the target power of the test, α is the assumed type I error rate, and $z_{q}$ is the qth quantile of a Gaussian distribution. Most of these are design parameters or constant for a given dataset. Interestingly, only σ and ρ differ between processing algorithms. Hence, the fraction of subjects needed using segmentation method 2 (S2) compared to segmentation method 1 (S1) can be expressed as

(21) $$R=100\frac{m_{\text{USLR}}}{m_{SS}}=100\frac{\sigma_{\text{USLR}}^{2}\left(1-\rho_{\text{USLR}}\right)}{\sigma_{SS}^{2}\left(1-\rho_{SS}\right)}.$$

We use the baseline and < 2 weeks observations to compute volume correlation and inter-subject variability. The test-retest data is not useful here as it scans subjects within the same session limiting the number of factors that explain inter-subject variability, such as hours of sleep or hydration.

**Fig. 10.**
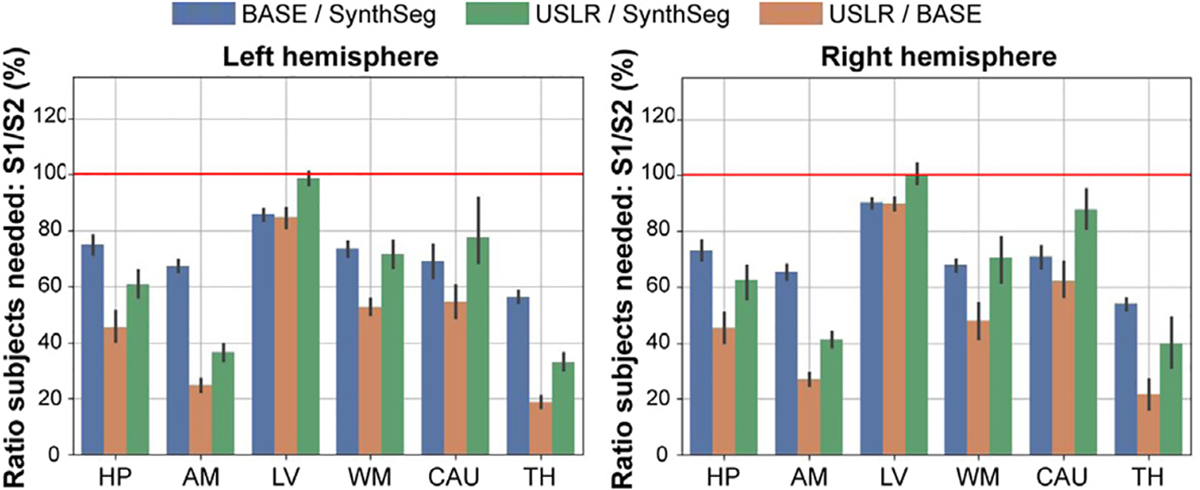
Power analysis showing the fraction of required subjects comparing segmentation methods S1 and S2. In blue, S1=BASE and S2=SynthSeg; in green S1=USLR and S2=SynthSeg; and in orange, S1=USLR and S2=BASE. A value < 100% indicates that S1 needs less subjects than S2 for given study specifications (power, target effect size, target power, etc.). The red line indicates that the same number of subjects are required between S1 and S2. BASE refers to longitudinal processing of timepoints using the baseline image as template. In the x-axis, we show the result of considering different subcortical region as primary outcome of the study.

In Fig. 10, we show the results for different brain regions as target endpoints. For example, in a study looking at right hippocampal differences using USLR, one would only need roughly 45% of subjects required compared to if cross-sectional SynthSeg were used or 60% of subjects compared to if the baseline image were used as template for longitudinal refinement. The reduction is even large for other relevant regions such as the thalamus or the amygdala. We hypothesise that the small reduction in the lateral-ventricle responds to the fact that the cross-sectional segmentations are simpler than in other regions, but sample sizes required using USLR are consistently lower than using SynthSeg.

#### 4.5.2. Linear mixed effects model

**Fig. 11.**
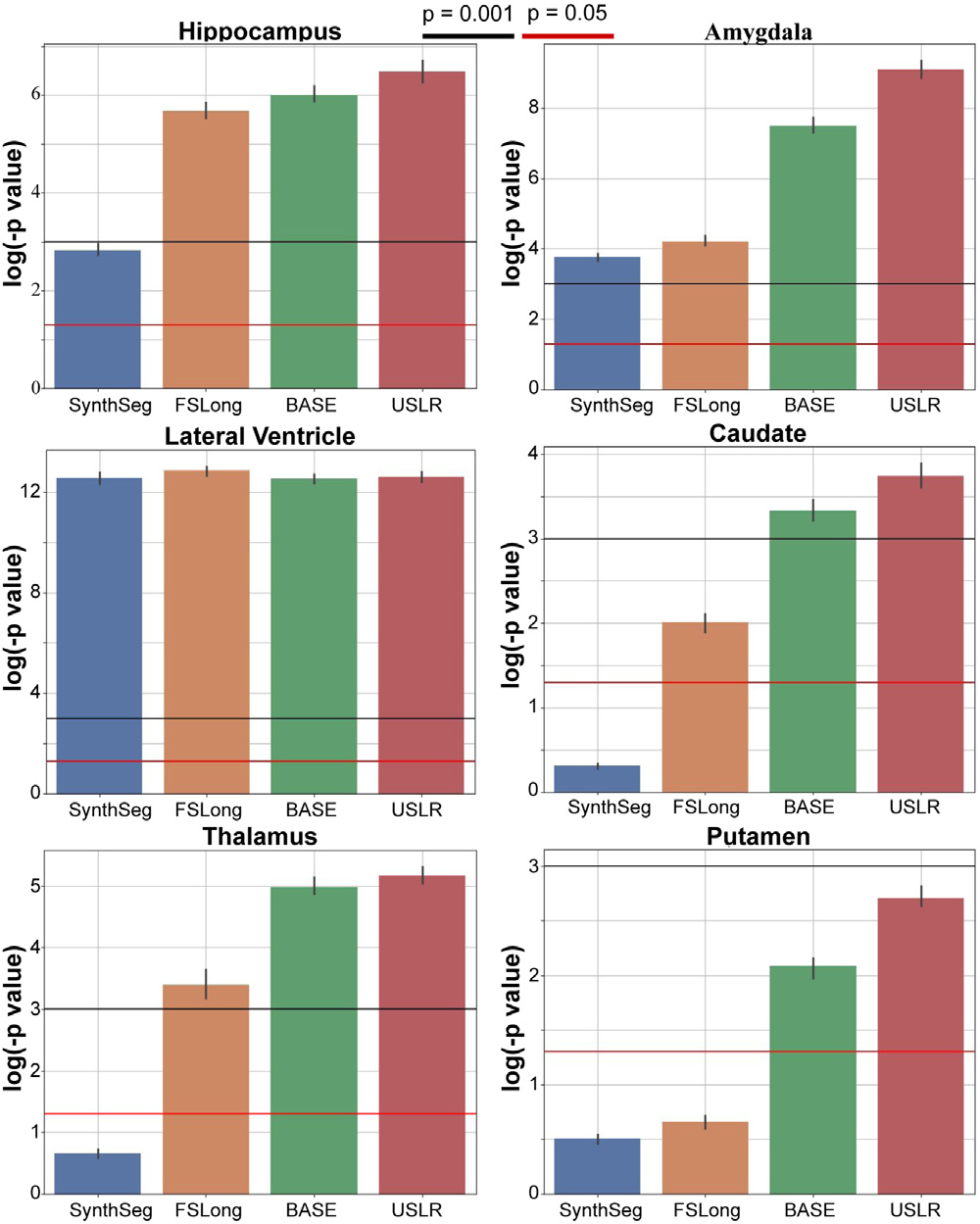
Linear mixed-effects model with random intercept and slope. We plot the log(p-value) of a contrast comparing time evolution between cognitively normal subjects and AD subjects. The bars represent the median value of N=1000 bootstrap sample. We compare three different segmentation methods: cross-sectional SynthSeg (blue), the longitudinal stream of Freesurfer (dark orange) and our USLR framework (green). Red line represents a p-value of $5\cdot 10^{-2}$ and the black line a p-value of $1\cdot 10^{-3}$.

**Fig. 12.**
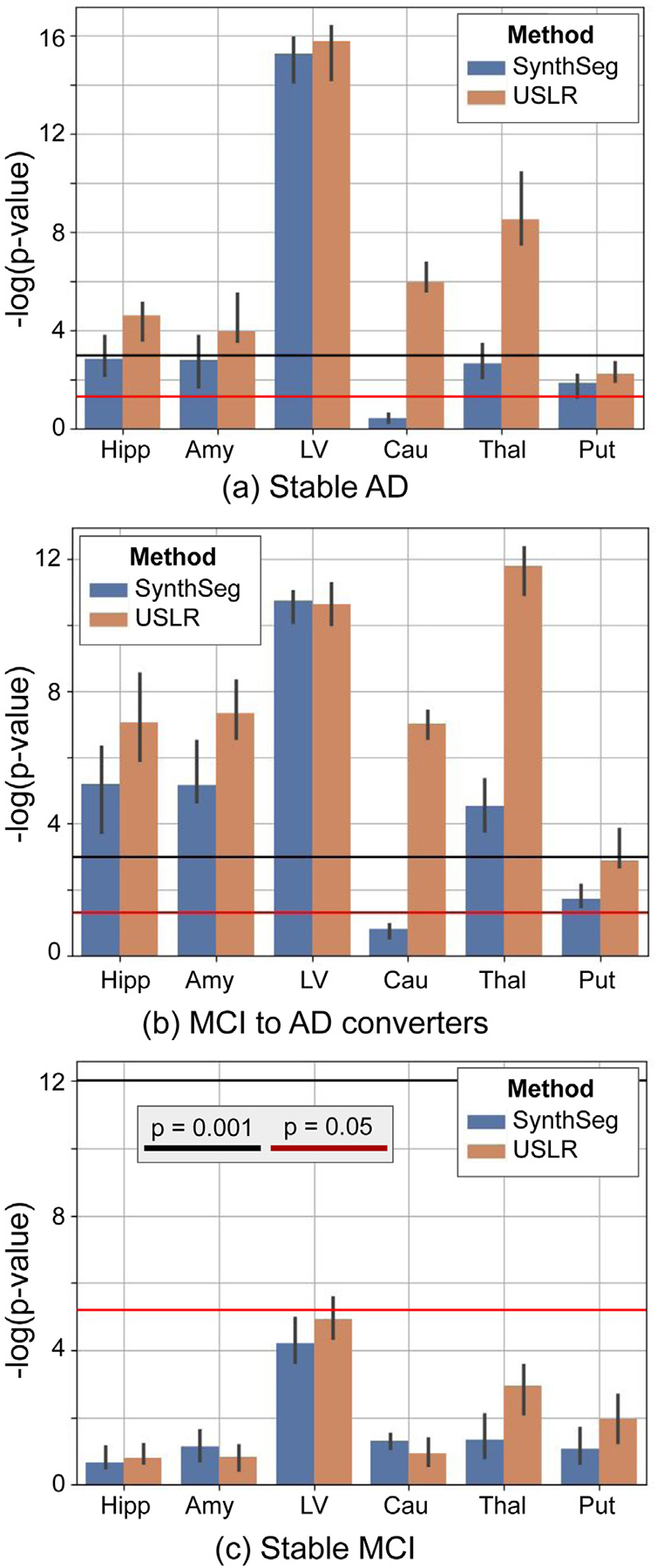
Linear mixed-effects model with random intercept and slope. The fixed effects features are time, age, sex, total intracranial volume and the interaction of the intercept and time with diagnosis (stable MCI, MCI to AD converter and stable AD). We plot the log(p-value) of a contrast comparing time evolution between cognitively normal subjects and the three diagnostic labels. The bars represent the median value of N=1000 bootstrap sample. We compare the USLR framework to SynthSeg. Red line represents a p-value of $5\cdot 10^{-2}$ and the black line a p-value of $1\cdot 10^{-3}$. Selected ROIs are: hippocampus (Hipp), amygdala (Amy), lateral ventricle (LV), caudate (Cau), thalamus (Thal) and putamen (Put).

Moving forward from subject-specific models to a population model, we test the statistical significance of the volumetric trajectories in describing the observations. We use a linear mixed-effects model including seven fixed effects (constant, time from baseline, age, sex, intracranial volume, diagnostic categories and interaction between diagnostic categories and time from baseline) and random intercept and slope. We employ a contrast on the interaction between diagnosis and time and compute the result on a bootstrap sample of size N. In Figs. 11 and 12, we selected the same subcortical regions and plot the resulting p-values with two different significance thresholds. In Fig. 11 we show the results on the MIRIAD dataset (N=1000), where the USLR volumetric trajectories appear more relevant than both cross-sectional (SynthSeg) and longitudinal (Freesurfer and using baseline as reference template) segmentations consistently for almost all subcortical regions.

In Fig. 12, we show the linear mixed-effect model results on ADNI subjects (N=50) to test the framework in a more diverse, heterogeneous and large-scale dataset. Here we include 4 diagnostic categories in the model, namely, stable healthy controls, stable MCI, MCI to AD converters and stable AD, and assess trajectory differences between patients and healthy controls. The USLR framework shows greater effect sizes in almost all subcortical ROIs tested. Moreover, for the chosen sample, the median effect size of regions such as the hippocampus, amygdala or thalamus only appear to be statistically significant $\left(p<1\cdot 10^{-3}\right)$ using USLR for stable AD patients (only p<0.05 using SynthSeg). Subjects that converted to AD during the time span of the study have the larger effect sizes (in median) for all ROIs and methods tested, except for the lateral ventricle. Lastly, subjects that remained at the MCI stage do not show any significant effects, as expected. Besides these significant effect sizes, we tested the predictive value of the rate of change to distinguish between diagnostic labels. We use a 5-fold cross-validation strategy with a random forest classifier and show that using USLR improves the overall classification (Section 5 in the supplementary material)

## 5. Discussion and conclusions

In this work, we introduce the USLR methodology, a framework for longitudinal registration of brain MRI scans. It capitalises on Bayesian inference and Lie algebra parameterisations of the spatial transforms to find unobserved deformation fields from each timepoint to a latent, unbiased subject-specific template. We define a probabilistic model in the space of deformations with pairwise registrations observed $\left(\left\{{ℛ}_{k}\right\}\right)$, and template to timepoint deformations hidden $\left(\left\{{𝒯}_{n}\right\}\right)$. This approach leads to a tractable, fast, and accurate framework that is independent of the registration algorithm (assuming that the deformation field can be parameterised in the log-domain). We could have modelled each $p\left(\left\{R_{k}\right\}|\left\{T_{n}\right\},\theta_{k}\right)$ with $\theta_{k}$ depending on the time distance between timepoints. This would, however, require a dedicated penalty function that relies on heuristics, as in Ashburner and Ridgway (2013), and the optimal regularisation will depend on the ultimate goal of the study. As discussed by Ashburner and Ridgway (2013), if the aim is to detect brain changes (as we do), less regularisation is preferred to reduce estimation bias at the expense of increased noise, because the result is non-linearly averaged over multiple registrations. Seemingly, it would be also possible to consider $\left\{{ℛ}_{k}\right\}$ as random variables conditioned to the original images, such that the uncertainties in the registration propagate to the estimates of $\left\{{𝒯}_{n}\right\}$. However, modelling the posterior distribution $p\left(\left\{{ℛ}_{k}\right\}|\left\{I_{n}\right\}\right)$ (e.g., via sampling) is often difficult and slow due to the high number of dimensions and strongly non-convex nature of the problem.

Importantly, USLR framework generalises to a variety of transformation models; here we use a rigid transform (USLR-rig), to account for global misalignment, followed by a non-linear stationary velocity fields that model local geometric differences between timepoints (USLR-diff). In both cases, the use of learning-based algorithms that are robust to acquisition differences (e.g., scanners, sequences, contrasts and resolutions) and that provide fast inferences makes the overall pipeline suitable for large scale datasets. This is a direct consequence of using SynthSeg to initialise the rigid registration part (Billot et al., 2023a) and SynthMorph (Hoffmann et al., 2022) as diffeormorphic registration algorithm. Furthermore, we have shown its benefits on a case-control study as compared to using cross-sectional processing pipelines.

Compared with existing similar approaches in the literature, we are able to compute unbiased and smooth time-varying longitudinal trajectories. For example, the authors in Joshi et al. (2004) focused only on the unbiasedness of the subject-specific template while the authors of Hadj-Hamou et al. (2016) focused only on computing a single stationary longitudinal trajectory with the baseline timepoint as reference. USLR computes a time-varying trajectory defined on an unbiased template while also outputs a single stationary trajectory useful in applications such as linear interpolation and extrapolation across time or DBM analyses. Moreover, the USLR pipeline output is successfully applied in downstream tasks and we have shown its benefits on a case-control study as compared to using cross-sectional processing pipelines.

One limitation of this work is the approximation of the composition of non-linear deformation fields by the summation of SVFs. As explored in the supplementary material (Section 3), the error incurred remained unnoticeable for the age and time span studied in this work, which is similar to typical follow up times present in clinical trials and observational studies in elderly adults. However, in contexts with large morphological changes with time, such as in brain development or post-surgery follow-up, the carried error by the BCH approximation may not hold and should be assessed, e.g., by visual inspection of the scans deformed with the fitted transformations.

We believe that this work serves as a proof-of-concept and that it opens up the use of USLR in multiple applications as well as extend USLR to integrate others. For example, we could easily include other Lie groups such as affine transforms (Li et al., 2023; Peter et al., 2018). Or, similarly to Cerri et al. (2023), we could accommodate segmentation maps in the USLR graph for a joint longitudinal Bayesian registration and segmentation method. As future directions, the USLR framework can be adapted to contexts with large follow-up changes by writing the objective function as the composition of deformation fields (rather than SVFs), and optimise them directly with numerical optimisation. This is however computationally much more expensive than the proposed solution. Finally, we plan to wrap the framework together with some other processing steps (inhomogeneity correction, segmentation, or normalisation to a template) and publish a comprehensive, well tested open-source pipeline available to the community.

## Supplementary Material

1

## Data Availability

The data used in this work is publicly available: MIRIAD and ADNI.

## Appendix A. Supplementary data

Supplementary material related to this article can be found online at https://doi.org/10.1016/j.media.2025.103662.
