# Supplementary material for: USLR: An open-source tool for unbiased and smooth longitudinal registration of brain MRI
Source: Med Image Anal. Author manuscript; Available in PMC 2026 May 13. (PMC13168522; doi:10.1016/j.media.2025.103662)
Supplement: 1 [file NIHMS2169052-supplement-1.pdf]

Contents lists available at ScienceDirect

Medical Image Analysis

journal homepage: www.elsevier.com/locate/media

Supplementary material of "USLR: an open-source tool for unbiased and smooth longitudinal registration of brain MRI"

Adrià Casamitjana[a,b,h,*], Roser Sala-Llonch[b,c,d], Karim Lekadir[e], Juan Eugenio Iglesias[f,g,1], for the Alzheimer's Disease Neuroimaging Initiative

[a] Universitat Politècnica de Catalunya. Barcelona, 08034, Spain
[b] Institut de Neurociències Department of Biomedicine, Faculty of Medicine, University of Barcelona, Barcelona, 08036, Spain
[c] Institut d'Investigacions Biomèdiques August Pi i Sunyer (IDIBAPS). Barcelona, 08036, Spain
[d] Centro de Investigación Biomédica en Red de Bioingeniería, Biomateriales y Nanomedicina (CIBER-BBN), Barcelona, 08036, Spain.
[e] Departament de Matemàtiques i Informàtica, Universitat de Barcelona, Artificial Intelligence in Medicine Lab (BCN-AIM), Barcelona, Spain
[f] Martinos Center for Biomedical Imaging, Massachusetts General Hospital and Harvard Medical School
[g] Computer Science and Artificial Intelligence Laboratory, Massachusetts Institute of Technology
[h] Centre for Medical Image Computing, University College London

1. Registration algorithms

The USLR framework relies on a generative model of the longitudinal trajectories (Eq. 6 from the main manuscript) that can be instantiated using different inverse-consistent deformation models. In this work, we use two types of transforms, namely rigid transforms and diffeomorphisms, computed using the following algorithms:

1.1. Rigid registration

Any rigid registration algorithm can be used together with USLR, as rigid transforms are inherently inverse consistent. Here, we use Procrustes analysis (PA, Goodall (1991)) to speed up the registration step – given that we need to compute $K = N \times (N-1)/2$ registrations. PA is a statistical shape analysis method that models images as point sets in a given space and minimises the distance between equivalent pairs of points. As in Iglesias (2023), we use the centroids for cortical and subcortical ROIs as points in the Euclidean space and find the rotation and translation that minimises the Euclidean distance between centroids:

$$\underset{\boldsymbol{U},\boldsymbol{t}}{\text{argmax}} \, ||\boldsymbol{C}_n(l) - \boldsymbol{t}_n - \boldsymbol{U}(\boldsymbol{C}_{n'}(l) - \boldsymbol{t}_{n'} + \boldsymbol{t})||_F,$$

The solution of this optimisation problem is the singular value decomposition of the point sets covariance centred at the origin: $(\boldsymbol{C}_n(l) - \boldsymbol{t}_n)(\boldsymbol{C}_{n'}(l) - \boldsymbol{t}_{n'})^\top$. It outputs the rotation matrix (U) and translation vector (t); the closed form expressions from Equation 8 in the main manuscript are used to compute the log-space parameters $\boldsymbol{v} = (\boldsymbol{q}, \boldsymbol{d})^\top$. A more compact form of the algorithm is detailed in Algorithm 1. Similar approaches can be found in the literature (Woods, 2003; Legouhy et al., 2023). On the one hand, Woods (2003) use Karcher's strategy to find a mean pose as a local minima in (semi-)Riemmanian manifolds. On the other hand Legouhy et al. (2023) also use ROI centroids as features to compute a global affine transform and a set of local affine transforms.

1.2. Non-rigid diffeomorphisms

The USLR framework can also be used together with any diffeomorphic registration algorithm that outputs stationary velocity fields (SVF). Here, we use SynthMorph (Hoffmann et al., 2022) to compute the observed SVF maps $\{\boldsymbol{R}(\boldsymbol{x})\}_k$ at 1/8 the image resolution $\boldsymbol{\psi}(\boldsymbol{x})$. This allows us to run USLR at a lower resolution, which is faster and produces smoother results. We use the exact same SynthMorph configuration to compute the registration between MNI and each subject's template.

SynthMorph is a learning-based registration framework outsourced with Freesurfer suite. It has been globally trained to compute pairwise symmetric diffeomorphisms for any pair of MRI contrasts and it adapts to different regularisation strength. Here we use the same default value as in the current implementation ($\lambda = 0.5$). To integrate the resulting SVFs and compute the deformation fields $\{\mathcal{R}_k(\boldsymbol{x})\}$, we use the "scaling and squaring" (Arsigny et al., 2006) algorithm followed by a rescaling layer with linear interpolation to upscale the deformation field to the original resolution.

*Corresponding author: Email: a.casamitjana@ub.edu

**Data used in preparation of this article were obtained from the Alzheimer's Disease Neuroimaging Initiative (ADNI) database (adni.loni.usc.edu). As such, the investigators within the ADNI contributed to the design and implementation of ADNI and/or provided data but did not participate in analysis or writing of this report. A complete listing of ADNI investigators can be found at: `http://adni.loni.usc.edu/wp-content/uploads/how_to_apply/ADNI_Acknowledgement_List.pdf`

Algorithm 1: Rigid registration

1. Given a pair of label maps S_n, $S_{n'}$ of images I_n and $I_{n'}$
2. Compute centroids $\boldsymbol{C}_n(l)$ and $\boldsymbol{C}_{n'}(l)$ for $l = 1, \ldots, L$.
3. Compute the translations:
   - $\boldsymbol{t}_n = \frac{1}{L}\sum_{l=1}^{L}\boldsymbol{C}_n(l)$,
   - $\boldsymbol{t}_{n'} = \frac{1}{L}\sum_{l=1}^{L}\boldsymbol{C}_{n'}(l)$
4. Shift point clouds to the origin:
   - $\hat{\boldsymbol{C}}_n(l) = \boldsymbol{C}_n(l) - \boldsymbol{t}_n$
   - $\hat{\boldsymbol{C}}_{n'}(l) = \boldsymbol{C}_{n'}(l) - \boldsymbol{t}_{n'}$
5. Compute the rotation matrix:

$$\boldsymbol{V}, \boldsymbol{\Delta}, \boldsymbol{S} = \mathrm{SVD}(\hat{\boldsymbol{C}}_n(l) \cdot \hat{\boldsymbol{C}}_{n'}^{\top}(l))$$
$$\boldsymbol{U} = \boldsymbol{S}\boldsymbol{V}^{\top}$$

6. Compute the final translation: $\boldsymbol{t} = \boldsymbol{t}_{n'} - \boldsymbol{U} \cdot \boldsymbol{t}_n$
7. Calculate log-space parameters $\boldsymbol{v} = (\boldsymbol{q}, \boldsymbol{d})^{\top}$

$$\boldsymbol{q} = \frac{\phi}{2\sin\phi}(U_{32} - U_{23}, U_{13} - U_{31}, U_{21} - U_{12})^{\top}$$
$$\boldsymbol{d} = \boldsymbol{P}^{-1}\boldsymbol{t}$$

where

$$\cos(\phi) = \frac{1}{2}(tr(\boldsymbol{U}) - 1)$$
$$\boldsymbol{P}^{-1} = \boldsymbol{I}_3 + 0.5\boldsymbol{Q} + \frac{(1 - \frac{\phi\cos\phi/2}{2\sin\phi/2})}{\phi^2}\boldsymbol{Q}^2$$

2. Linear program

Here, we present in more detail the inference algorithm presented in Section 2.5 of the manuscript, which can be re-written as a linear program as follows:

$$\begin{aligned} \text{minimize } & \boldsymbol{c}^T\tilde{\boldsymbol{y}} \\ \text{s. t. } & \boldsymbol{A}_1^T\tilde{\boldsymbol{y}} \leq 0, \\ & \boldsymbol{A}_2^T\tilde{\boldsymbol{y}} \leq 0, \\ & \boldsymbol{A}_3^T\boldsymbol{y} \leq -\boldsymbol{R}^j(x), \\ & \boldsymbol{A}_4^T\boldsymbol{y} \leq \boldsymbol{R}^j(x), \end{aligned}$$

where:

- $\tilde{\boldsymbol{y}} = [D_0^j(\boldsymbol{x}), \boldsymbol{y}^T]^T$, is the $(K+N+1)\times 1$ vector of unknown latent variables, concatenating the deviation associated to the regularisation term, $D_0^j(\boldsymbol{x})$, and $\boldsymbol{y}$, both defined below.
- $\boldsymbol{y} = [D_1^j(\boldsymbol{x}), ..., D_K^j(\boldsymbol{x}), T_1^j(\boldsymbol{x}), ..., T_N^j(\boldsymbol{x})]^T$ is a $(K+N)\times 1$ vector concatenating the K absolute deviations of the model, $D_k^j(\boldsymbol{x})$ (defined below), and the latent transforms to estimate, $T_n^j(\boldsymbol{x})$.
- $\boldsymbol{c} = [\boldsymbol{1}_{K+1}^T, \boldsymbol{0}_N^T]^T$, where $\boldsymbol{1}_{K+1}$ and $\boldsymbol{0}_N$ are the all-one and all-zero vectors with dimensions $(K+1)\times 1$ and $N \times 1$, respectively.
- $\boldsymbol{A}_1 = [-1, \boldsymbol{0}_K^T, -\boldsymbol{1}_N^T]$ is a $(K+N+1)\times 1$ vector.
- $\boldsymbol{A}_2 = [-1, \boldsymbol{0}_K^T, \boldsymbol{1}_N^T]$.
- $\boldsymbol{A}_3 = [-\boldsymbol{I}_K, -\boldsymbol{W}]$, where $\boldsymbol{I}_K$ is the $K \times K$ identity matrix.
- $\boldsymbol{A}_4 = [-\boldsymbol{I}_K, \boldsymbol{W}]$.

By using vector $\boldsymbol{c}$, this linear program effectively minimises the model deviations and it is equivalent to the problem of minimising C_{ℓ_1} in Equation 14 from the manuscript. The inequality constraints effectively force the deviations $D_k^{\xi_j}(\boldsymbol{x})$ to be positive and equal to:

$$D_0^j(\boldsymbol{x}) = \frac{b_T}{b_Z}|\sum_{n=1}^{N}\boldsymbol{T}_n^j(\boldsymbol{x})|,$$
$$D_k^j(\boldsymbol{x}) = |\boldsymbol{R}_k^j(\boldsymbol{x}) - \sum_{n=1}^{N}\boldsymbol{W}_{kn}\boldsymbol{T}_n^j(\boldsymbol{x})|, \quad \forall k \in (1, \cdots, K).$$

The solution is then simply the second part (last N elements) of the vector $\boldsymbol{y}$ and can be obtained using well-established linear programming algorithms, such as HiGHS (Huangfu and Hall, 2018) (used here) or interior-point methods (Karmarkar, 1984; Andersen and Andersen, 2000).

3. Effect of Baker-Campbell-Hausdorff (BCH) truncation

The probabilistic model of USLR operates in the Lie algebra parameterisation of the deformation fields. This could be directly optimised but it is computationally demanding both in terms of memory and time. Instead, we truncate the BCH formula to approximate the composition of deformation fields by the sum of log-space parameterisations. This step linearalises the probabilistic model which can now be solved analytically (Gaussian model of the registration noise) or using a linear program (Laplacian model of the registration noise) reaching a global minimum in both cases.

To empirically evaluate the error accumulated by the truncation of the BCH formula, we optimised Eq.11 without the BCH approximation, i.e., composing the fields exactly. This is a non-convex function with generally many local maxima, which we optimise iteratively with the LBFGS algorithm. This is a much slower process that converge to different solutions depending on the initialisation. We used two different initialisation strategies: first, using a zero-field; and second, using the solution of the BCH approximation. In Fig. 1, we show the difference between the SVFs computed exactly and truncating the BCH formula on all MIRIAD subjects.

Firstly, we see that our model's approximation is already a good solution of the problem, as it tends to converge to similar solutions when initialised with the zero field; moreover, it tends to remain stable when initialised with our model's ℓ_2 solution. Crucially, we note that direct optimisation of the deformation fields is computationally more intensive. Secondly, we see that

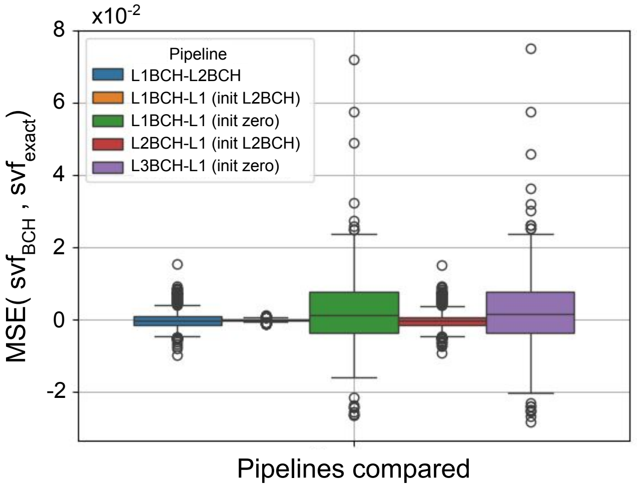

Fig. 1: Boxplot showing the difference between non-truncated and truncated SVFs incurred when making the BCH approximation of the field composition with the sum. For the truncated version, we compare ℓ_1 (L1BCH) and ℓ_2 optimisation (L2BCH). For the non-truncated version, we use a Laplacian (ℓ_1) likelihood and two initialisation schemes: zero-field and using the L2BCH solution. The L1BCH is not used to reduce the computation time overhead.

ℓ_1 and ℓ_2 optimisation of the truncated BCH produce very similar results. Nonetheless, due to the long tails of the registration residue measured empirically (shown in Fig. 4 in this letter), a Laplacian model seems better suited for this problem compared to the Gaussian model.

4. Probabilistic model

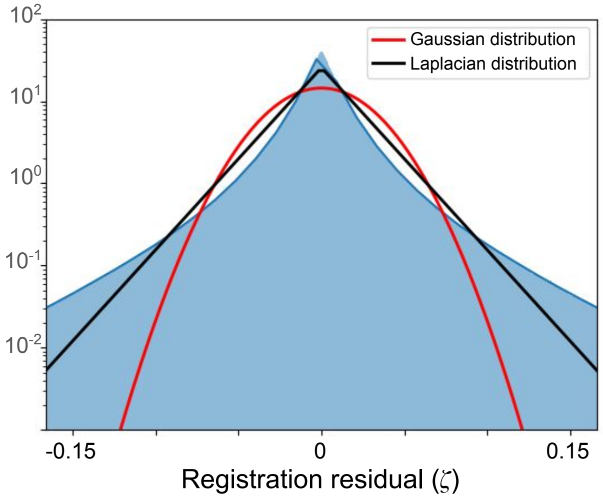

Fig. 2: Empirical registration error from our model in (Eq. 4) computed on the whole MIRIAD sample. The y-axis is in the log-scale. A Gaussian (red) and Laplacian (black) distribution with the observed standard deviation are overlaid.

The probabilistic model in Eq. 4 in the main text is defined in the space of deformations and relates observed pairwise registrations ($\{\mathcal{R}_k\}$), and latent, unobserved template to timepoint deformations ($\{\mathcal{T}_n\}$). Using the BCH approximation, this model is linear in the log-space parameterisation of the deformation fields. In such scenario, a Laplacian distribution is chosen to model the registration residue in the log-domain, $\boldsymbol{\zeta}$ mainly for two reasons: (*i*) it is more robust against registration errors in the observations, as also shown in our previous paper (Casamitjana et al., 2022); and (*ii*) because we empirically observe (Fig. 2) that the computed registration errors have long tails which are better modelled using a Laplace distribution.

5. Subject-specific stationary trajectories

We use the MIRIAD dataset to further asses the longitudinal subject-specific signatures estimated by USLR, and compare our method to other commonly used approaches: (1) 2 TP, which registers the first (used as reference template) and last timepoints (i.e., $I_1 \rightarrow I_N$); and (2) BASE, that registers all timepoints to the baseline image (used as reference template) so that the trajectory between the i-th and the $(i + 1)$-th timepoints is the composition $I_i \rightarrow I_1 \rightarrow I_{i+1}$. The longitudinal signature of 2 TP is directly computed from the SVF between the first and last timepoints; the signature using the BASE method is computed by a linear fit on the computed SVF maps from each timepoint to the template using time-to-baseline as independent variable.

5.1. *Estimation error*

To assess the goodness-of-fit of the linear fit, we compute the estimated error (mm) between the integrated estimated SVF signatures and the real computed deformations between the template and each timepoint. We report the results in Figure 3 averaged over four different brain ROIs and stratified by diagnostic categories. We see that the linear approximation on USLR trajectories is much better than the other two approaches, consistently for different brain ROIs. This result is also indicative of the smoothness of the USLR trajectories.

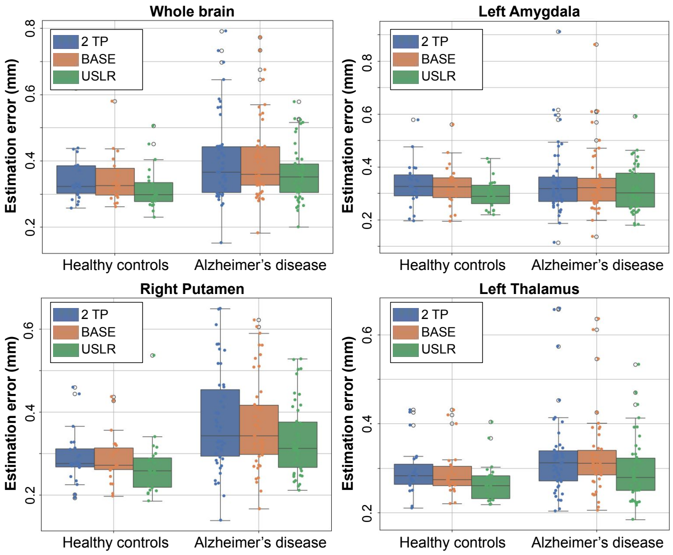

Fig. 3: Estimated error (mm) of the linear fit on the SVF trajectories for three different methods: registering first and last timepoints (2 TP, in blue), using the baseline image as reference (BASE, orange) and our proposed method (USLR, green). We stratify the result for healthy controls and Alzheimer's disease (AD) patients in different brain ROIs.

5.2. *Smoothness*

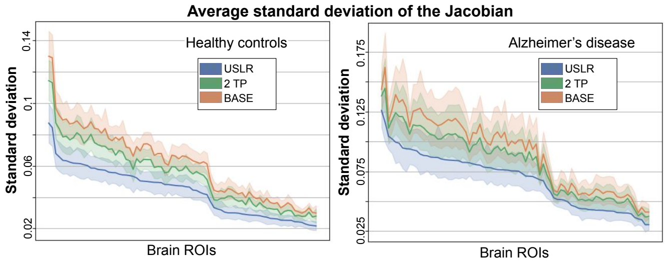

Fig. 4: Comparison of the standard deviation of the Jacobian determinant map within multiple cortical and subcortical brain ROIs between three different approaches: registering first and last timepoints (2 TP, in green), using the baseline image as reference (BASE, orange) and our proposed method (USLR, blue). Each brain ROI is defined on the subject-specific template segmentation. Brain ROIs are ordered from larger to lower standard deviations according to the USLR method. The results are stratified between healthy controls and Alzheimer's disease patients to see the difference in smoothness.

We evaluate the smoothness of the trajectories using Jacobian determinant signature map of each subject and computing the standard deviation across all voxels belonging to each brain ROI as defined by the segmentation of the subject-specific template. The result is shown in Figure 4, were we consistently see that the USLR method has lower standard deviations than other methods for all brain ROIs and diagnostic categories. A lower standard deviation is a measure of increased trajectory smoothness within each brain ROI.

5.3. *TBM and DBM analyses*

We statistically compare the Jacobian determinant ("tensor-based morphometry", Hua et al. (2008)) between the BASE and USLR methods. We resample each subjects' Jacobian to MNI space using the deformation field computed registering the reference template of each approach to the MNI2009a nonlinear template. We then use a simple t-test between healthy controls and Alzheimer's disease subjects and report the significant t-values at $p-$value < 0.05 corrected for multiple comparisons using FDR (Figure 5. Figure 7 from the manuscript shows the USLR result; here, in Figure 5, we report the BASE results and the comparison between USLR and BASE t-values. Despite a similar pattern is found in USLR and BASE – significant expansion of ventricular areas and shrinkage of gray matter cortical (e.g, entorhinal cortex) and subcortical (e.g., mediodorsal thalamic nuclei) structures–, we see that the statistical significance of USLR is higher in the majority of spatial locations.

6. Rate of change prediction

To go beyond statistical analyses, we used a simple random forest classifier to test whether the computed group trajectory differences could be used to predict the diagnostic category. For each subject, we fit a linear model to the trajectory of 6 different subcortical ROIs on each hemisphere and extract the intercept and slope as input features to the classifier. Hence, we extract a total of 24 features per subject.

We perform two different binary classification tasks: first, predict AD subjects from healthy controls and second, predict

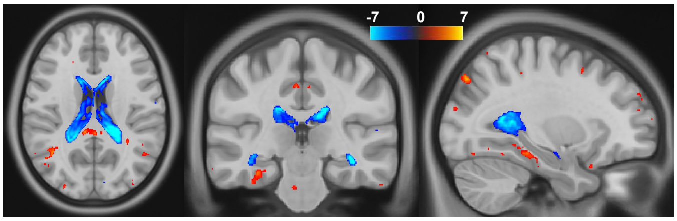

(a) BASE

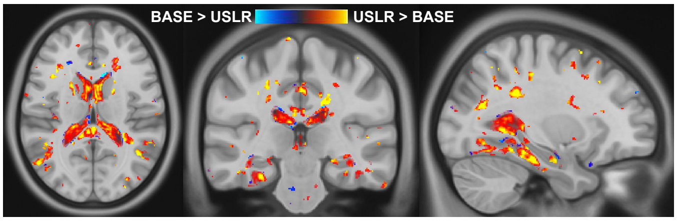

(b) USLR-BASE

Fig. 5: T-test results on the Jacobian determinant between healthy controls and Alzheimer's disease subjects. We show (a) the t-values thresholded at $p = 0.05$ corrected for multiple comparisons using FDR of the BASE method and (b) the comparison between the USLR and BASE t-values.

MCI subject that will convert to AD (progressive MCI or pMCI) from those that will remain on the same status (stable MCI or sMCI). In each case, we split the sample into train and test using a 5-fold strategy and evaluate 5 different metrics: accuracy, f1-score, precision, recall and area under the ROC curve (auc).

Table 1: Results of a random forest classifier in a 5-fold validation study. We evaluate two different tasks: prediction of AD subjects from healthy controls (CN vs. AD) and prediction of MCI sujects that will progress to AD from those that remain stable on the same status (sMCI vs pMCI). Performance values are the mean and ± standard deviation from the 5 folds.

Task	metric	SynthSeg	USLR
CN vs. AD	accuracy	0.86 (±0.07)	**0.89 (±0.06)**
	f1-score	0.88 (±0.06)	**0.90 (±0.06)**
	precision	0.89 (±0.1)	**0.91 (±0.08)**
	recall	0.88 (±0.04)	**0.89 (±0.08)**
	auc	0.86 (±0.08)	**0.89 (±0.06)**
sMCI vs. pMCI	accuracy	0.67 (±0.07)	**0.73 (±0.04)**
	f1-score	0.64 (±0.13)	**0.72 (±0.08)**
	precision	0.74 (±0.11)	**0.77 (±0.04)**
	recall	0.59 (±0.18)	**0.69 (±0.14)**
	auc	0.68 (±0.07)	**0.72 (±0.04)**

References

Andersen, E. D., Andersen, K. D., 2000. The mosek interior point optimizer for linear programming: an implementation of the homogeneous algorithm. In: High performance optimization. Springer, pp. 197–232.

Arsigny, V., Commowick, O., Pennec, X., Ayache, N., 2006. A log-euclidean framework for statistics on diffeomorphisms. In: Medical Image Computing and Computer-Assisted Intervention–MICCAI 2006: 9th International Conference, Copenhagen, Denmark, October 1-6, 2006. Proceedings, Part I 9. Springer, pp. 924–931.

Casamitjana, A., Lorenzi, M., Ferraris, S., Peter, L., Modat, M., Stevens, A., Fischl, B., Vercauteren, T., Iglesias, J. E., 2022. Robust joint registration of multiple stains and mri for multimodal 3d histology reconstruction: Application to the allen human brain atlas. Medical image analysis 75, 102265.

Goodall, C., 1991. Procrustes methods in the statistical analysis of shape. Journal of the Royal Statistical Society: Series B (Methodological) 53 (2), 285–321.

Hoffmann, M., Billot, B., Greve, D. N., Iglesias, J. E., Fischl, B., Dalca, A. V., 2022. Synthmorph: learning contrast-invariant registration without acquired images. IEEE transactions on medical imaging 41 (3), 543–558.

Hua, X., Leow, A. D., Parikshak, N., Lee, S., Chiang, M.-C., Toga, A. W., Jack Jr, C. R., Weiner, M. W., Thompson, P. M., Initiative, A. D. N., et al., 2008. Tensor-based morphometry as a neuroimaging biomarker for alzheimer's disease: an mri study of 676 ad, mci, and normal subjects. Neuroimage 43 (3), 458–469.

Huangfu, Q., Hall, J. J., 2018. Parallelizing the dual revised simplex method. Mathematical Programming Computation 10 (1), 119–142.

Iglesias, J. E., 2023. A ready-to-use machine learning tool for symmetric multimodality registration of brain mri. Scientific Reports 13 (1), 6657.

Karmarkar, N., 1984. A new polynomial-time algorithm for linear programming. In: Proceedings of the sixteenth annual ACM symposium on Theory of computing. pp. 302–311.

Legouhy, A., Callaghan, R., Azadbakht, H., Zhang, H., 2023. Polaffini: Efficient feature-based polyaffine initialization for improved non-linear image registration. In: International Conference on Information Processing in Medical Imaging. Springer, pp. 614–625.

Woods, R. P., 2003. Characterizing volume and surface deformations in an atlas framework: theory, applications, and implementation. NeuroImage 18 (3), 769–788.
